# Frontotemporal-TDP and LATE Neurocognitive Disorders: A Pathophysiological and Genetic Approach

**DOI:** 10.3390/brainsci13101474

**Published:** 2023-10-18

**Authors:** Genaro Gabriel Ortiz, Javier Ramírez-Jirano, Raul L. Arizaga, Daniela L. C. Delgado-Lara, Erandis D. Torres-Sánchez

**Affiliations:** 1 Department of Philosophical and Methodological Disciplines, University Health Sciences Center, University of Guadalajara, Guadalajara 44340, Jalisco, Mexico; daniela.delgadolara@gmail.com; 2 Postgraduate Gerontology Program, University Health Sciences Center, University of Guadalajara, Guadalajara 44340, Jalisco, Mexico; 3 Neurosciences Division, Western Biomedical Research Center, Mexican Social Security Institute, IMSS, Guadalajara 44340, Jalisco, Mexico; ramirez_jirano@hotmail.com; 4 Public Health Department, School of Medicine, University of Buenos Aires, Buenos Aires C1121ABG, Argentina; raul.luciano.arizaga@gmail.com; 5 Departamento Académico de Formación Universitaria, Ciencias de la Salud, Universidad Autónoma de Guadalajara, Zapopan 45129, Jalisco, Mexico; 6 Department of Medical and Life Sciences, University Center of la Cienega, University of Guadalajara, Ocotlan 47820, Jalisco, Mexico

**Keywords:** frontotemporal lobar degeneration, transactive response DNA-binding protein 43, limbic age-related encephalopathy, progranulin

## Abstract

Frontotemporal lobar degeneration (FTLD) belongs to a heterogeneous group of highly complex neurodegenerative diseases and represents the second cause of presenile dementia in individuals under 65. Frontotemporal-TDP is a subgroup of frontotemporal dementia characterized by the aggregation of abnormal protein deposits, predominantly transactive response DNA-binding protein 43 (TDP-43), in the frontal and temporal brain regions. These deposits lead to progressive degeneration of neurons resulting in cognitive and behavioral impairments. Limbic age-related encephalopathy (LATE) pertains to age-related cognitive decline primarily affecting the limbic system, which is crucial for memory, emotions, and learning. However, distinct, emerging research suggests a potential overlap in pathogenic processes, with some cases of limbic encephalopathy displaying TDP-43 pathology. Genetic factors play a pivotal role in both disorders. Mutations in various genes, such as progranulin (GRN) and chromosome 9 open reading frame 72 (C9orf72), have been identified as causative in frontotemporal-TDP. Similarly, specific genetic variants have been associated with an increased risk of developing LATE. Understanding these genetic links provides crucial insights into disease mechanisms and the potential for targeted therapies.

## 1. Biology of TDP-43

### 1.1. Structure of TDP-43

Transactive response DNA-binding protein 43 (TDP-43) is a highly conserved, predominantly nuclear protein encoded by the TAR-DNA-binding protein (TARDBP) gene found on chromosome 1. The TDP-43 name comes from its discovery when it was identified as a human trans-activation response element (TAR) binding protein [1]. TDP-43 belongs to the family of heterogeneous nuclear ribonucleoproteins (hnRNPs), which are proteins that primarily interact with pre-messenger transcripts. The TARDBP gene encodes a single isoform of 414 amino acids. Structurally, TDP-43 has two ribonucleic acid (RNA)-binding domains (RRM1 and RRM2, where RRM stands for RNA recognition motif) and a glycine-rich C-terminal region used to interact with other proteins such as hnRNP A1/A2 and C1. This region also contains a prion-like domain (rich in glutamine/asparagine) that plays an important role in TDP-43 aggregation [2]. Also, TDP-43 has nuclear export (NES) and nuclear localization (NLS) sequences necessary for its passage from the nucleus to the cytoplasm and *vice versa.*

### 1.2. Physiological Functions of TDP-43

Described initially as a DNA-binding protein, TDP-43 is now known to regulate nucleic acid metabolism through different mechanisms, primarily:

#### 1.2.1. Nuclear Functions: Modulation of Pre-mRNA Splicing by TDP-43

In 2001, a study highlighted the participation of TDP-43 in splicing exon 9 of the pre-mRNA encoded by the cystic fibrosis transmembrane conductance regulator (CFTR) gene [3]. Subsequently, the use of the high-throughput sequencing of RNA isolated by the crosslinking immunoprecipitation (HITS-CLIP) technique allows the identification of RNA bound to a protein, revealing that TDP-43 interacts with a large part of the nervous system transcriptome (central nervous system (CNS)) (>6000 RNA). Interestingly, most of these RNAs involve essential cellular processes such as neuronal development, axonal guidance, and synaptic activity [4]. Furthermore, many of these transcripts show splicing changes in FTLD with amyotrophic lateral sclerosis (ALS) patients and TDP-43-deficient mouse models [5,6]. TDP-43 binds mainly at the level of introns and 3′UTR regions (untranslated regions) on sequences preferably rich in UG repeats, although this feature is not essential for interaction. Thus, TDP-43 can interact with its own mRNA’s 3′UTR region to modulate its translation capacity. This autoregulatory mechanism is essential in maintaining the amount of TDP-43 in the cell at a physiological level [7].

#### 1.2.2. Nuclear Functions: Regulation of Micro-RNAs

Less than 5% of the RNA produced inside cells codes for proteins. It is now known that the remaining 95%, called non-coding RNAs (ncRNAs), play an essential role in regulating gene expression within the CNS, both under physiological and pathological conditions [8]. Micro-RNAs (miRNAs) are part of these ncRNAs, and their function is to repress the expression of the target mRNAs by pairing directly with the complementary sequences to them [9]. The synthesis of miRNAs involves several steps between the nucleus and the cytoplasm and mainly involves two RNase IIIs called Drosha and Dicer. In fact, after the transcription of the primary miRNA (pri-miRNA), the latter is cleaved by the Drosha protein and forms a sequence called pre-miRNA [10]. This pre-miRNA is then transported to the cytoplasm where Dicer will mature it, resulting in a functional 22 nucleotide miRNA [11].

The hypothesis that TDP-43 could participate in the regulation of miRNAs was raised by its presence within the Drosha complex and its association at the level of perichromatin fibrils, where pri-miRNAs are born [12]. In agreement with this first observation, studies have shown deregulation of the expression of various miRNAs, such as let-7b, miR-663, or miR-132 [12]. Most of these deregulations result from a direct interaction between TDP-43 with miRNAs or their precursors. However, during *in vitro* neuronal differentiation, TDP-43 was shown to modify Drosha stability, suggesting a potential impact on all; in addition, increased Dicer expression was also observed upon TDP-43 repression in a human neuronal cell line model [13]. Together, these data support the critical role of TDP-43 in miRNA metabolism.

#### 1.2.3. Nuclear Functions: Regulation of the Expression of Long Non-Coding RNAs

Among the other ncRNAs, long non-coding RNAs (lncRNAs) are involved in numerous cellular mechanisms, particularly at the transcriptional and post-transcriptional levels. Recently, the deregulation of these lncRNAs has been demonstrated, particularly in pathologies related to aging, including neurodegenerative diseases [14]. In this context, TDP-43 has been shown to affect the expression of several lncRNAs such as MALAT1 (associated with SC35 domains called speckles) and NEAT1_2 (involved in the formation of paraspeckles) [6]. In fact, in the brain of FTLD/ALS patients, TDP-43 binds more to these lncRNAs, and increased expression of NEAT 1_2 was observed in motor neurons of ALS patients, compared to healthy individuals [15]. Given the importance of specks and parameters in RNA metabolism, dysregulation of these lncRNAs could contribute to the pathophysiology of FTLD/ALS [16]. However, although the direct interaction of TDP-43 with these lncRNAs has been established, the mechanisms underlying their dysregulation and how this contributes to neurodegeneration remain to be determined [17]. Although TDP-43 is primarily nuclear, it is also credited with several functions in the cytoplasm including regulation of mRNA stability, transport, and translation.

#### 1.2.4. Cytosolic Functions: Stabilization of mRNA

Transactive response DNA-binding protein 43 was first identified as a regulator of the stability of the mRNA encoding neurofilament lumen (NF-L) and histone deacetylase HDAC6 [18]. Studies demonstrated that 3′UTR regions of mRNAs, which play a crucial role in mRNA stability mediated by RNA-binding proteins (RBPs), were a preferential target of TDP-43 [4,13]. As a result, other targets of TDP-43, such as β-adducin (Add2) [19], vascular endothelial growth factor A (VEGFA), GRN [20], or even interleukin-6 (IL-6) [21], were discovered.

#### 1.2.5. Cytosolic Functions: Transport of mRNA

Within polarized cells such as neurons, transporting mRNAs to axons and dendrites is a fundamental process in maintaining neuronal activity and synaptic plasticity [22]. Evidence suggests that TDP-43 is involved in this process. First, a study on cultures of primary motor neurons demonstrated the transport of TDP-43 along axons. Interestingly, this study also showed that TDP-43 co-localized with the survival motor neuron protein (SMN) and fragile-X mental retardation protein (FMRP), both of which are known to play a role in mRNA transport within specific structures called mRNA granules [23]. Transport of positive TDP-43 mRNA granules along axons in motor neurons derived from human inducible pluripotent stem cells (iPSCs) was then confirmed. Among the mRNAs present within these granules, the NF-L mRNA, whose stability is regulated by TDP-43, stood out significantly.

**Figure 1:**
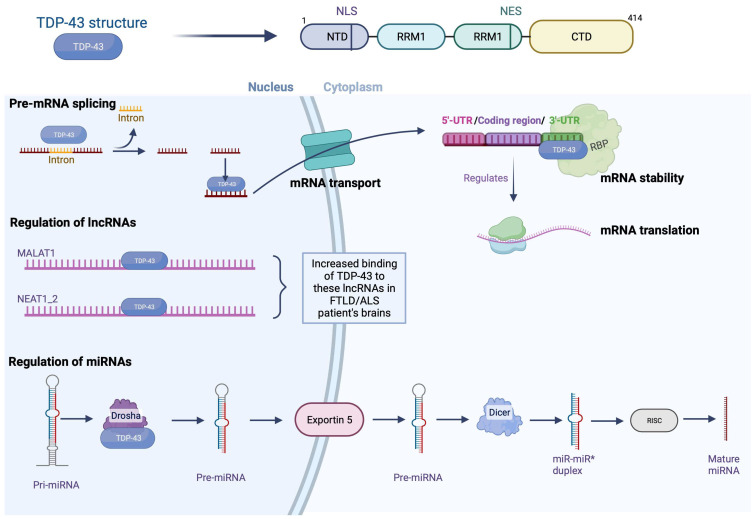
Physiological functions of TDP-43. TPD-43 is a nuclear protein with an N-terminal domain (NTD), two RNA recognition motifs (RRM1 and RRM2), and a long C-terminal domain (CTD). TDP-43 regulates the metabolism of nucleic acid by different mechanisms such as pre-miRNA splicing (TDP-43 binds to introns and 3′UTR regions); it binds to mRNA formed and transports it from the nucleus to the cytoplasm through pores. In the cytoplasm, it gives stability to mRNA by binding to 3′UTR region and is believed to regulate the mRNA translation. In addition, TDP-43 has been shown to bind to long non-coding RNAs such as MALAT1 and NEAT1_2 in neurodegenerative diseases; binding is increased in the brain of FTLD/ALS patients. Finally, it helps in the regulation of miRNAs; the primary miRNA (pri-miRNA) is cleaved by Drosha, and the pre-miRNA is formed, which is transported to the cytoplasm by Exportin 5, where it will mature by Dicer, followed by RISC, and result in a functional miRNA of 22 nucleotides. Nuclear export sequence (NES); nuclear localization sequence (NLS); RNA-binding protein (RBP). Created with Biorender.com.

Furthermore, the presence of TDP-43 mutations within these iPSCs induces impaired anterograde granule transport, indicating a direct role for TDP-43 in axonal mRNA transport [24]. TDP-43 was also bound to mRNAs encoding proteins related to synaptic function in adult mouse brains. These data, associated with the localization of TDP-43 at the level of the axonal terminals, therefore indicate that TDP-43 plays a role in the transport of RNAs toward the extensions of neurites (Figure 1).

#### 1.2.6. Cytosolic Functions: Regulation of mRNA Translation

One of the emerging functions of TDP-43, directly related to its role in mRNA transport to distal regions, is the regulation of local mRNA translation. The first proof of their involvement was highlighted in 2008 when a team observed a TDP-43 translocation at the dendrite level after potassium chloride stimulation within rat hippocampal neurons [25]. Furthermore, this study indicated that TDP-43 could repress mRNA translation within mRNA granules. Proteomic analyzes confirmed these results by revealing the interaction between TDP-43 and the translation machinery [26]. A study using cell cultures subjected to oxidative stress also demonstrated the presence of TDP-43 within inactive ribosome-enriched polysomes [27]. More recently, the use of an ALS model in *Drosophila* has described a regulation of the translation of the Futsch/22C10 microtubule-associated proteins 1B (MAP1B) and Rac1 proteins by TDP-43 [28,29]. These proteins are involved in neuronal functioning, modulating the formation of dendrites and the architecture of neuromuscular junctions. Therefore, TDP-43 dysregulation could play a crucial role in the pathophysiology of FTLD. A study of the translational profile in murine models of ALS also indicates the deregulation of two mRNAs bound by TDP-43 at the motor neuron level, encoding the DDX58 and MTHFSD proteins, respectively [30] (Figure 1).

### 1.3. Pathophysiology of TDP-43

Aggregated forms of TDP-43 exhibit several abnormal changes including hyperphosphorylation, ubiquitination, and N-terminal proteolysis [31]. At the histopathological level, the lesions observed are mainly of the cytoplasmic (NCIs) and neuritic (due to dystrophic neurites (DN)) type, and show immunoreactivity for both TDP-43 and p62 (a protein linked to autophagy). In some cases, usually hereditary, neuronal intranuclear inclusions (NII) are also present. Moreover, other types of lesion that are not ubiquitin-positive have also been demonstrated. Among them can be distinguished diffuse cytoplasmic markings (called pre-inclusions), neuritic markings, and cytoplasmic inclusions within oligodendrocytes (glial cytoplasmic inclusion (GCI)) [32].

Regarding the distribution of lesions, these are commonly found in the frontal and temporal cortex and in the dentate *gyrus* of the *hippocampus*. However, many other subcortical regions may also be affected [33]. Depending on the distribution of the inclusions within the cortical layers, and the nature of the inclusions, four histopathological subtypes are defined; each of these subtypes may be partially correlated with a specific clinical syndrome or genetic mutation [32].

## 2. The Different Histopathological Subtypes of FTLD-TDP

### 2.1. FTLD-TDP Type A

Type-A FTLD-TDP is distinguished by the presence of numerous DNs and cytoplasmatic aggregates at the level of the second layer of the neocortex. A moderate number of NCI granular inclusions are also present within the dentate *gyrus* [34]. In addition, GCIs are found in the white matter and subcortical regions such as the *striatum*, *thalamus*, and *substantia nigra.* At the clinical level, the cases affected by this type of TDP-43 pathology generally present with behavioral variant of frontotemporal dementia (bvFTD) or β-amyloid protein experimental model (APPnfv).

### 2.2. FTLD-TDP Type B

Type-B FTLD-TDP cases show NCI lesions in the outer and deep cortical layers with relatively little DN or NII [34]. Immunohistological analyzes also reveal a significant number of pre-inclusions and neurites, mainly in the superficial layer of the cortex. The presence of NCI lesions in lower motor neurons is also one of the specificities of type B, even in the absence of clinical signs of ALS. Glia is also affected, with many GCIs in the medullary white matter and the spinal cord—most cases have an FTLD/ALS clinical phenotype present with FTLD-TDP type B.

### 2.3. FTLD-TDP Type C

Type-C FTLD-TDP is characterized by long neuritic lesions, mainly in the outer layer of the cortex [34]. Some NCI lesions can also be found, both in the cortex and in the *hippocampus.* The presence of NII and GCI is sporadic. Type C is the most common pathologic subtype in patients with semantic variant primary progressive aphasia (svPPA).

### 2.4. FTLD-TDP Type D

This pathologic subtype’s hallmark is abundant lentiform NII lesions and short neuritic lesions in the neocortex [34]. Various subcortical regions (*basal ganglia*, *thalamus*, *hippocampus*, *cerebellum*, etc.) can also be heterogeneously affected. This histopathological presentation is rare since it is only found in cases carrying a mutation in the valosin-containing protein (VCP) gene.

**Figure 2:**
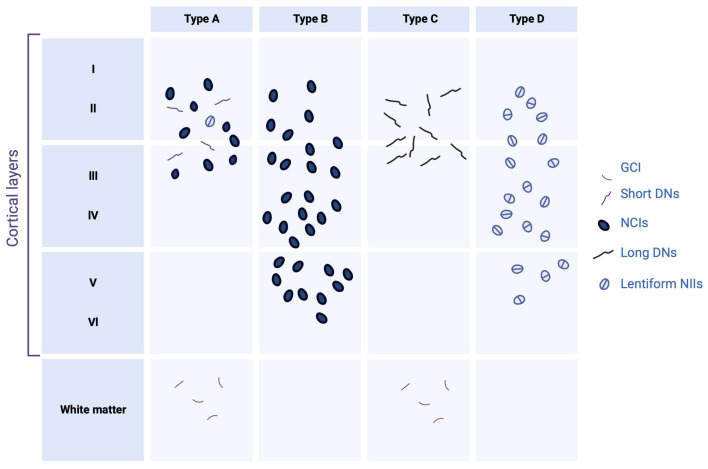
Histopathological subtypes of FTLD-TDP. Type A is characterized mainly by short dystrophic neurites (DNs) and neuronal cytoplasmic inclusions (NCIs) in the superficial neocortical layers. Type B is characterized by NCI in the superficial and deep neocortical layers. Type C is characterized by long DN lesions in the superficial layers. Type D is characterized by lentiform neuronal intranuclear inclusions (NIIs). Glial cytoplasmic inclusion (GCI) presents in white matter only in types A and B. Created with Biorender.com.

The etiology of most cases of FTLD and ALS is still unclear; yet, these cases are related to each other by the presence of TDP-43 pathology characterized by cytoplasmic delocalization, presence of truncated forms, aggregation, and post-translational modifications of this protein (Figure 2).

## 3. TDP-43 Mutations

### 3.1. Post-Translational Modifications

In brain tissue from patients with FTLD and ALS, TDP-43 hyperphosphorylation and ubiquitination represent one of the main features of the pathology. Indeed, these two modifications preferentially label cleaved, aggregated, and insoluble forms of TDP-43 [35,36]. Immunohistological studies in human tissue indicate that specific TDP-43 lesions, particularly pre-embeddings, do not label positively with ubiquitin [37]. These results suggest that ubiquitination would occur later in the pathophysiological process.

### 3.2. Phosphorylation

There are 64 potentially phosphorylatable sites on TDP-43, including 41 serine residues, 15 threonine residues, and 8 tyrosine residues. Among these sites, 29 are phosphorylated by CK1 protein kinase (casein kinase 1) [38]. Experimentally, Western blot characterization of tissues from patients with FTLD and ALS underscores that the pS379, pS403/404, and pS409/410 sites are the most characteristic of TDP-43 pathology [35]. Furthermore, the pS409/410 site represents the most-studied phosphorylation site. The truncated or cytoplasmic forms are phosphorylated in many cell and animal models [39,40,41]. Overexpression of wild-type TDP-43 leads to aggregates of phosphorylated TDP-43 proteins similar to those seen in FTLD or ALS cases [42,43]. Phosphorylated forms of TDP-43 exhibit a longer half-life than unphosphorylated forms, suggesting that phosphorylation may inhibit the ubiquitin–proteasome system (UPS)-mediated degradation of TDP-43 and thus promote its aggregation [44]. In addition, the mutation of the serine residues 409 and 410 in alanine allows it to mimic a non-phosphorylated state of the protein, which abolishes the toxicity of TDP-43 when overexpressed in *Caenorhabditis elegans.*

Similarly, inhibition of protein kinases involved in TDP-43 phosphorylation, such as cell division cycle-related protein kinase 7 (CDC7) or tau tubulin kinase (TTBK), also reduces the toxicity induced by TDP-43 phosphorylation [45,46]. There is also evidence supporting a protective effect of phosphorylation [47]. Phosphorylation appears to be a relatively early event in the development of TDP-43 pathology. However, although phosphorylation is closely related to aggregation, it is unclear whether this is a cause or a consequence of aggregation. Indeed, studies indicate that TDP-43 phosphorylation is not essential for TDP-43-induced cleavage, aggregation, or toxicity [48,49]. These results, combined with the fact that the physiological role of TDP-43 phosphorylation remains undetermined, make the use of therapeutic tools directed at this modification complex.

### 3.3. Ubiquitination

Ubiquitination is another mitochondrial permeability transition (MPT) that affects TDP-43. Indeed, TDP-43 is modified by lysine-linked polyubiquitin chains 48 or 63 [50]. Furthermore, the presence of ubiquitinated forms of TDP-43 (truncated forms and complete forms) in various animal and cellular models suggests that the UPS is the primary pathway for TDP-43 degradation [41,42]. The fact that proteasome inhibition promotes TDP-43 accumulation and aggregation supports this hypothesis [41]. TDP-43 has a relatively long half-life of between 12 and 34 h in most cell models, while that of carboxy-terminal fragments (CTFs) is around 4 h [51]. A different interaction with the UPS can explain this difference since CTFs do not have the NLS and are mainly located in the cytoplasm [39]. Because of its lability, the accumulation of CTF is quite surprising and could result from decreased degradation with age [52]. Although TDP-43 ubiquitination is not as widely studied as phosphorylation, several studies have focused on it. Therefore, ubiquitination of TDP-43 by ubiquitin ligases ubiquitin-conjugating enzyme (UBE2E) promotes the transition of TDP-43 to an insoluble state, but does not promote its degradation [53].

In contrast, Parkin-mediated ubiquitination induces the translocation of TDP-43 into the cytosolic compartment and may enhance its degradation [50]. Similarly, attempts to promote TDP-43 degradation by blocking the release of polyubiquitin chains have led to mixed results. Therefore, inhibition of ubiquitin-specific peptidase (USP14) in mouse embryonic fibroblasts would promote TDP-43 clearance by maintaining ubiquitin chains [54]. Repression of deubiquitinase UBPY expression (by ubiquitin isopeptidase Y) exacerbates TDP-43 toxicity in *Drosophila*, despite the retention of ubiquitin chains [53].

### 3.4. Truncation

**Figure 3:**
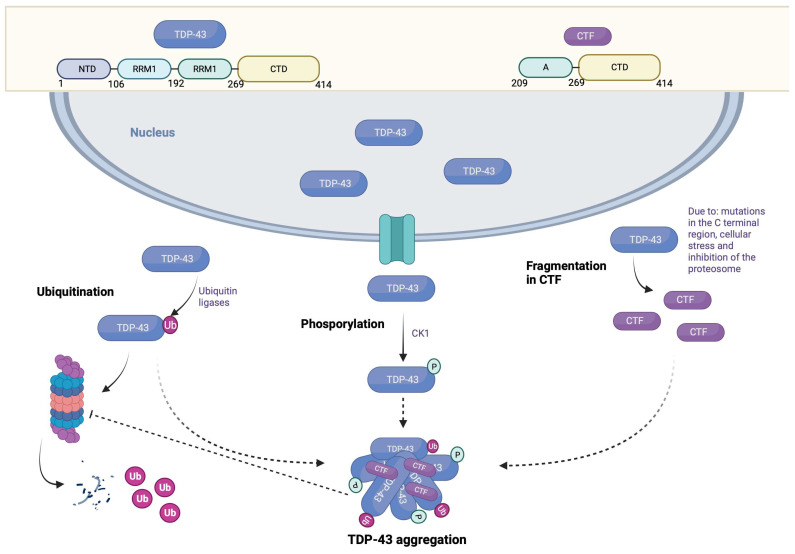
Post-translational modifications of TPD-43. The principal post-translational modifications found in TDP-43 protein inclusion associated with pathogenic alterations observed in FTLD/ALS patients are ubiquitination, hyperphosphorylation, and aberrant cleavage. The image shows (a) the ubiquitination of TPD-43 through activation of the ubiquitin–proteasome system (UPS) which affects its protein concentration; (b) TDP-43 being phosphorylated by casein kinase 1 (CK1) which may inhibit the UPS and promotes TDP-43 aggregation; and (c) TDP-43 fragmentation in carboxy-terminal fragments (CTFs). Altogether, these have been proposed to promote TDP-43 aggregation. Created with Biorender.com.

TDP-43 CTF between 20 and 25 kDa constitutes one of the biochemical signatures of FTLD-TDP. TDP-43 proteolysis is an event mediated by several mechanisms, including mutations of TDP-43 in its C-terminal region [55], cellular stress [48,56], and inhibition of the proteasome. Due to the absence of NLS in the generated fragments, the latter accumulate in the cytoplasm and act as pro-aggregating factors thanks to the presence of the prion-like domain [2]. These CTFs could serve as the basis for inclusion formation by sequestering the complete TDP-43 protein. However, there is currently no evidence that TDP-43 cleavage directly modulates TDP-43 toxicity and even appears to have a protective effect in some cases [57]. TDP-43 toxicity seems to require preserving the ability to interact with RNAs, which is absent in CTFs [58]. An *in vitro* study indicates that *de novo* CTF production is insufficient to induce TDP-43 aggregation, and a second event is required for this [51]. It is essential to highlight that TDP-43 truncation seems necessary for its degradation under physiological conditions. Therefore, blocking TDP-43 proteolysis as a therapeutic tool is not the best option (Figure 3).

### 3.5. TDP-43 Relocation

Loss of nuclear localization associated with aggregation in the cytoplasm or the neurites is one of the hallmarks of FTLD-TDP. Although most TDP-43 inclusions are extranuclear, we have seen that some are located in the nucleus (NII). TDP-43 typically localizes to euchromatic regions of the nucleoplasm, especially perichromatin fibrils, where transcription and splicing occur [12]. Therefore, even when the inclusions are nuclear, TDP-43 is still considered to be mislocalized. Delocalization of TDP-43 in the cytoplasm can be modulated by several factors, including ALS-associated TARDBP mutations, cellular stress [59], and impaired TDP-43 degradation. Various cellular and animal models have been developed to assess how the delocalization of TDP-43 in the cytoplasm contributes to neurodegeneration. The form of TDP-43 mutated in the NLS has been widely used. To induce a mainly cytoplasmic localization of TDP-43 [39,41], this delocalization is accompanied by aggregation of the mutated form, which is also capable of recruiting native TDP-43 proteins; however, several studies have shown that the wild-type form of TDP-43 was sufficient to induce toxicity, even in the absence of inclusions [60]. Regardless, decreasing the cytoplasmic localization of TDP-43 appears to reduce toxicity *in vivo*; however, it must be emphasized that these studies do not directly address the nucleocytoplasmic transport of TDP-43, but other mechanisms, such as autophagy. The question arises whether it is the native or malformed form that is responsible for the toxicity induced by TDP-43 in the cytoplasm.

### 3.6. Aggregation

Intraneuronal aggregates of TDP-43 characterize FTLD-TDP. The presence of protein aggregates is a phenomenon found in many neurodegenerative diseases. However, these inclusions’ protective or harmful role is still highly debated. Indeed, regarding the correlation between aggregation and all the pathological modifications found (delocalization, truncation, phosphorylation, ubiquitylation, etc.), it is not easy to designate aggregation as the primary pathophysiological mechanism. TDP-43 is no exception to this observation, and many studies point to differential roles for TDP-43 aggregation in pathology. Therefore, although transgenic animal models expressing the full-length form of TDP-43 show some TDP-43 inclusions, these are relatively rare and do not correlate with the phenomenon of neurodegeneration [61].

Prevention of TDP-43 inclusion formation does not appear to reduce toxicity in a cell model. Nevertheless, it is essential to underline that these studies are based on the presence of visible aggregates of TDP-43. Still, these aggregates may be only the final step involving different TDP-43 species ranging from simple monomers to mature aggregates via oligomers. This hypothesis is supported by studies indicating that the complete form of TDP-43 can form oligomeric structures with amyloid properties. These oligomers are found both in the brain of FTLD-Parkinson disease dementia (PDD) patients and in transgenic mice, and can induce the aggregation of other amyloid peptides such as Aβ [62].

## 4. TDP-43 Considerations about Being a Prion Protein

The spread of TDP-43 represents an emerging mechanism in the pathophysiology of FTLD-TDP. This hypothesis is based in particular on the presence of a prion-like domain in the C-terminal part of TDP-43 [2], but also on the fact that there is a hierarchical progression of the pathology in FTLD-PDD [63]. However, this is less well established than for tau in Alzheimer’s Disease (AD), and a correlation between TDP-43 pathology progression and duration of disease progression could not be established. The pro-aggregative role of this prion-like domain is remarkably supported by two facts:

1. Most of the TDP-43 mutations found in ALS target the C-terminal part.
2. CTFs, which essentially contain this prion domain, appear to promote aggregation *in vivo* [40].

**Figure 4:**
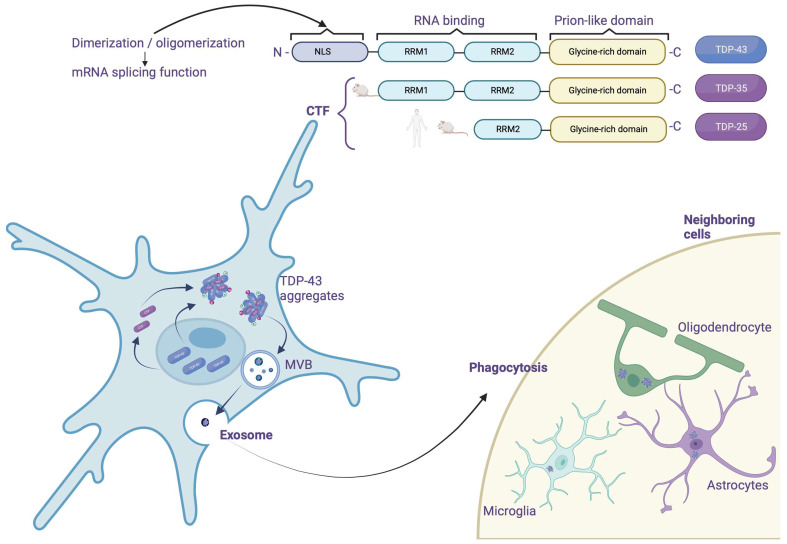
Mechanism of propagation of TDP-43. TDP-43 possesses a long C-terminal domain that is rich in glycine. This region is a prion-like domain where most of the TDP-43 mutations found in ALS occur and the part that promotes aggregation. In humans, TDP-43 is fragmented to TPD-25, and in rats it has been shown to be fragmented to TDP-25 and TDP-35. The proposed spread from one cell to another cell is via exosomes or microvesicles (MVB). Created with Biorender.com.

Although there is currently no *in vivo* evidence, several studies suggest that TDP-43 can propagate and induce aggregation of native TDP-43 proteins. First, recombinant TDP-43 aggregates, previously formed *in vitro*, can induce the accumulation of endogenous TDP-43 proteins within HEK293T cells [64]. Similarly, the addition of insoluble TDP-43 proteins from FTLD/ALS brains into SH-SY5Y cells induces the formation of ubiquitinylated and phosphorylated endogenous TDP-43 aggregates. Furthermore, it has been shown that TDP-43 oligomers could spread from one cell to another via exosomes or microvesicles, results which suggest that prion properties attributed to TDP-43 might play a role in the pathophysiology of FTLD; nonetheless, they would need to be confirmed *in vivo* (Figure 4).

## 5. FTLD-TDP Genetics

### 5.1. Granular Precurson (GRN) Gene

The first mutations in the GRN gene were demonstrated in 2006. More than 69 pathological mutations in the progranulin gene have been identified [65], representing a number of cases from 5 to 20% of FTLD of familial origin. Most of the mutations affecting the GRN gene are of the nonsense type and lead to reduced gene expression, suggesting that haploinsufficiency is the primary pathological mechanism. Carriers of these mutations show a 50% reduction in the amount of mRNA and around 33% reduction in protein.

### 5.2. Progranulin (GRN) Structure

Like microtubule-associated protein tau (MAPT), the GRN progranulin gene is located on chromosome 17 at position q21. This gene encodes a 68.5 kDa protein composed of 593 cysteine-rich amino acids. The full-length form of the protein contains 7.5 well-conserved domains, each consisting of a repeat of 12 cysteine motifs (also called granulin motifs) separated by junction regions. Once in the extracellular medium, various proteases can cleave progranulin at the level of these regions to generate fragments ranging from 6 to 25 kDa, called granulins [66].

### 5.3. Expression in the CNS of Progranulin

Progranulin is a secreted protein expressed in many tissues and cell types throughout the body. In the brain, its expression is low during the early stages of development and increases with age. Regarding its distribution in different cell types, progranulin is found mainly in neurons and microglia, although it may be present in small amounts in astrocytes and ependymocytes [67]. Its expression within microglia is significantly increased under pathological conditions [68,69].

### 5.4. Modulation of Expression by Transmembrane Protein 106B (TMEM106B)

The penetrance of mutations in the GRN gene is incomplete, so knowing the factors that regulate the expression of this gene is essential from a therapeutic point of view. A genome-wide association study (GWAS) of FTLD-TDP cases identified the gene encoding TMEM106B as a modulator of pathology in patients with or without a GRN gene mutation. The genetic variability of TMEM106B notably affects the penetrance of the disease in cases carrying a mutation in the GRN gene, but also the age of onset of the first symptoms [70]. In addition, people with the risk allele show a lower plasma level of progranulin [70,71]. The TMEM106B gene encodes a transmembrane protein capable of associating with progranulin in endolysosomes [72]. Regarding the underlying mechanism, a study in cells indicates that the increase in the expression of TMEM106B promotes the intracellular accumulation of progranulin [73]. This would imply the sequestration of progranulin within the lysosomes, which would block its release into the extracellular space and/or reduce its degradation.

In summary, the genes that are associated with FTLD-TDP are *GRN*, *MAPT*, *C9ORF72*, *TARDBP*, and *VCP*, although other genes not yet properly identified for FTLD-TDP but that may be related are *HNRNPA2B1*, *SQSTM1*, *UBQLN2*, and *TREM2* [74], as can be seen in Table 1.

### 5.5. Biological Function of Progranulin

Progranulin involves many physiological and pathological mechanisms, including cell proliferation, inflammation, and metabolic diseases. On the other hand, its role in the brain remains largely unknown despite intense research since the discovery of its involvement in FTLD in 2006.

### 5.6. Progranulin Neuronal Function and Growth of Neural Tracts

**Figure 5:**
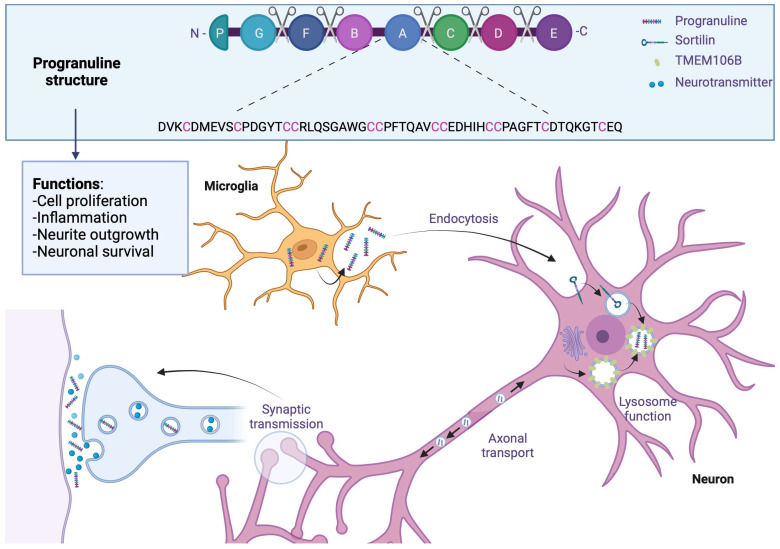
Progranulin in frontotemporal lobar degeneration. Progranulin contains 7.5 conserved domains, consisting of a repeat of 12 cysteine motifs separated by junction regions where some proteases will be able to cleave progranulin. Progranulin is found mainly in neurons and microglia, where its expression is increased in FTLD; through endocytosis, progranulin is bound to the sortilin receptor and sequestrated in lysosomes which are associated with TMEM106B, which would block its release into extracellular space and reduce its degradation. In adult brains, progranulin is associated with synaptic plasticity; patients with progranulin gene mutation reported an increase in synaptic vesicles and extrasynaptic secretion. Created with Biorender.com.

Regarding the role of progranulin as a growth factor at the peripheral level, many studies have focused on the potential role it plays in neuronal growth and survival. The expression of progranulin or granulin (E) has been shown to promote neuronal survival and neurite outgrowth in rat primary neuronal cultures [92]; furthermore, a study in zebrafish underscores the vital role of progranulin in the development of motor neurons. Repression of the expression of progranulin homologs in zebrafish induces a reduction in axonal growth. In murine primary neuron cultures, treatment with progranulin shows an increase in the development of neuronal tracts via glycogen synthase kinase-3beta (GSK-3β). In contrast, using small interfering RNA (siRNA) directed against progranulin decreases the neurite tree in primary cultures of rats [93]. The addition of recombinant progranulin in GRN -/- mice has been shown to restore neurite outgrowth. In a progranulin-deficient mouse model, analysis of dendrite size at the CA1 pyramidal cell level (*hippocampus*) once again supports the role of progranulin in neurite outgrowth: given the low expression of progranulin during embryonic development, its role in neuritic growth is probably more associated with synaptic plasticity in the adult brain (Figure 5).

### 5.7. Synaptic Plasticity

Evidence suggests that progranulin can modulate synaptic biology. Thus, an increase in the number of synaptic vesicles and their probability of secretion has been observed in cultures of rat hippocampal neurons deficient in progranulin. These results were confirmed in the brains of FTLD patients with a mutation in the GRN gene [93]. Although another study indicates the opposite by highlighting a decrease in the number of these vesicles in GRN -/- mice, all these data support the role of progranulin in synaptic function. In addition, dysregulation of miRNA expression targeting proteins involved in synaptic biology was demonstrated in FTLD-PDD patients carrying a GRN gene mutation [94]. In addition, the stimulation of neuronal activity would promote the recruitment of progranulin at the synaptic level and its synaptic and extrasynaptic secretion. A link between loss of progranulin and synaptic dysfunction has been described; in fact, a study has shown an increase in inhibitory synapse removal in mice whose progranulin expression has been repressed in microglia. This phenomenon would be mediated in particular by increased complement expression resulting from increased microglial activation in humanized immune system (HIS) mice [95]. Progranulin can also bind to the sortilin receptor, which is known to bind neuropeptides such as neurotensin and neurotrophic factor pro-nerve growth factor (NGF). Progranulin binding to the sortilin receptor causes its endocytosis and rapidly leads to its degradation via the lysosomal pathway. However, no signaling pathway appears to be activated [96,97], suggesting only a role for sortilin in regulating extracellular progranulin concentration [98]. Progranulin would also be involved in the stress response, which would play a protective role (Figure 5).

### 5.8. Microglial Functions

Due to the overexpression of progranulin in pathological conditions, several studies have focused on its role in microglia. Thus, a study observed an excessive secretion of cytokines in primary cultures whose microglial progranulin expression is repressed [99]. Furthermore, this excess of cytokines appears to be cytotoxic. A study conducted in a similar mouse model also showed an increase in complement production [95]. In contrast, using siRNAs directed against progranulin decreases cytokine production in human fetal microglial cells after lipopolysaccharide (LPS) stimulation [100]. Progranulin also seems to act as a chemoattractant for microglial cells, suggesting a role in the recruitment of these cells during CNS damage.

### 5.9. Progranulin on TDP-43 Aggregation

Transactive response DNA-binding protein pathology is characterized by phosphorylated forms of the complete TDP-43 protein and C-terminal fragments in the brain tissue of FTLD patients. The relationship between progranulin haploinsufficiency and the development of this pathology is unknown. Although several studies *in vitro* observe proteolysis and aggregation of TDP-43 during progranulin depletion [56,101,102], these results are still controversial. Furthermore, no pathological modification of TDP-43 is found after the repression of the progranulin gene in zebrafish or human cell lines [103]. Progranulin-deficient mouse models show highly variable phenotypes; thus, while some models are distinguished by TDP-43 phosphorylation and/or delocalization [104,105], others do not show signs of TDP-43 pathology, and this occurs even at older ages. However, a recent study demonstrated that certain granulins, particularly granulin (E), would promote TDP-43 toxicity in different animal models. An accumulation of this fragment is also observed in brain regions of patients affected by the TDP-43 pathology [106].

### 5.10. Neuropathology and Associated Clinical Signs

The onset of symptoms is later than for MAPT mutations, with a mean between 59 and 65 years. However, the duration of the evolution is similar, with an average of 9 years. Singularly, there is significant heterogeneity in the clinical symptoms observed in patients, even in individuals with identical mutations or belonging to the same family. Most cases present with clinical signs of bvFTD or svPPA, often accompanied by Parkinsonian syndrome. An association with ALS is found very rarely [107]. The main feature observed on imaging in patients carrying a GRN mutation is the presence of asymmetric brain atrophy [108].

### 5.11. White Matter Abnormalities

**Figure 6:**
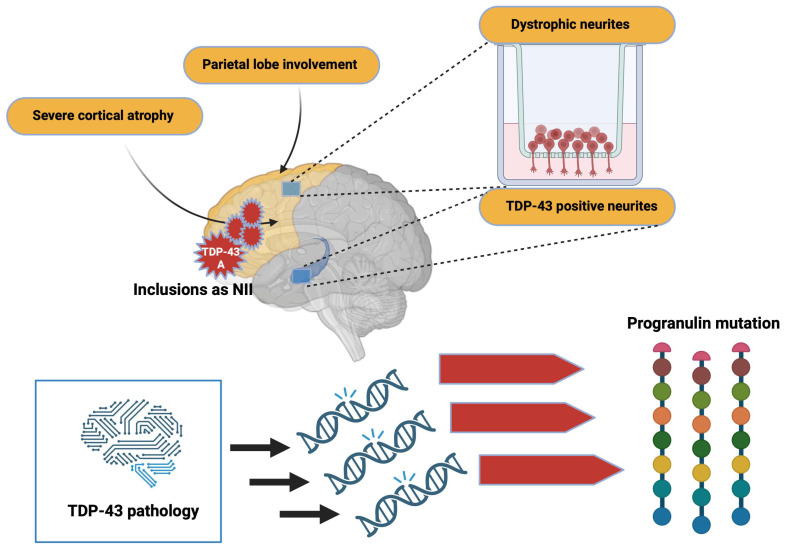
Alterations due to TDP-43 pathology. TDP-43 pathology induces mutations that trigger important changes in progranulin. In addition, inclusions by TDP-43 can alter brain morphology. Created with Biorender.com.

Severe cortical atrophy and parietal lobe involvement are also found in these patients. From a neuropathological point of view, TDP-43 type-A inclusions are mainly found. Inclusions can also appear as NII and are primarily located in the frontal cortex and *striatum* [109]. Dystrophic neurites are frequently found in the superficial cortical layers, and minor TDP-43-positive neurites are found in the CA1 *hippocampus* [110,111]. Furthermore, morphometric studies indicate that the TDP-43 pathology affects specific neuronal circuits in patients with a progranulin mutation. The asymmetric distribution of TDP-43 lesions, distributed mainly in the left hemisphere, makes it possible, in particular, to differentiate TDP-43 pathology related to GRN mutations from that sometimes found in patients with AD (Figure 6).

### 5.12. Nucleotide C9ORF72

In 2011, a GGGGCC hexanucleotide repeat was identified in a non-coding region of the C9ORF72 gene as the major common genetic cause of FTLD and ALS [112]. In fact, although variable, the expansion size is approximately 23 repeats in a healthy individual, while it can reach several thousand repeats in affected patients [112]. Although the exact minimum number of repeats to induce pathology is still not well determined, most studies consider the presence of more than 30 repeats pathological [112,113]. Currently, 20 to 40% of familial cases of ALS and FTLD, respectively, are explained by a C9ORF72 mutation [114]. Given the significant involvement of C9ORF72 in FTLD and ALS, research has focused on understanding how the GGGGCC repeats lead to the phenomenon of neurodegeneration.

### 5.13. Pathophysiological Mechanisms Related to GGGGCC Repeats

Three hypotheses are proposed to explain hexanucleotide repeats’ pathological mode of action.

**Figure 7:**
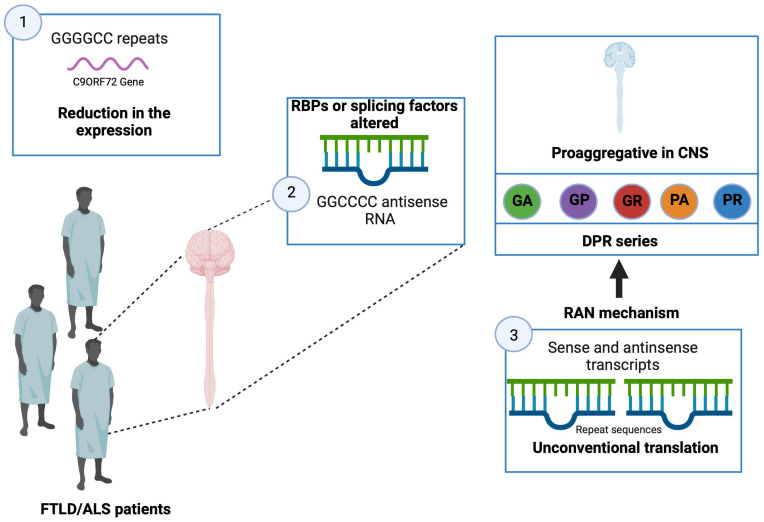
Alterations in patients with FTLD/ALS. In patients with FTLD/ALS, a reduction in the expression of the C9ORF72 gene is present, and alteration in RBPs as well as in splicing factors and changes in transcription processes are observed. Created with Biorender.com.

1. The presence of many repeats could cause a reduction in the expression of the C9ORF72 gene leading to a loss of its physiological function. It would seem that the expression of the C9ORF72 gene is reduced in patients carrying this type of mutation [112].
2. It has been shown that brain and spinal cord tissue from FTLD/ALS patients is not distinguished by the presence of nuclear foci composed of GGGGCC RNA but also GGCCCC antisense RNA [115,116], similar to the mechanism described in myotonic dystrophy type I (DM1) [117]; these foci could sequester certain RBPs or splicing factors and lead to a loss of their physiological functions.
3. The transcripts (sense and antisense) produced from the repeated sequences would be the target of an unconventional translation mechanism that does not depend on the presence of an ATG codon. This mechanism, called translation-associated repeat not initiated by ATG (RAN), would be responsible for the production of a series of dipeptides (dipeptide repeat, DPR; glycine-alanine, GA; glycine-proline, GP; glycine-arginine, GR; proline-alanine, PA; and proline-arginine, PR) [116]. These DPRs, located throughout the entire CNS, have the characteristic of being pro-aggregative, and, therefore, could participate in the neurodegenerative process [116]. Several studies carried out in cell culture models of *Drosophila* demonstrate the toxicity of these DPRs [115,118] (Figure 7).

## 6. Clinical Presentation and Neuropathology

As with mutations affecting the progranulin gene, there is some clinical heterogeneity between members of the same family. Thus, the age of onset of symptoms is highly variable and ranges from 21 to 83 years, with a mean of 50 years [114]. As with GRN mutations, variants of the TMEM106B gene may influence disease onset in patients with an expansion in the C9ORF72 gene [119]. The disease lasts 1 to 22 years, with a mean of 8 to 9 years [114]. The most common phenotype found in patients carrying a mutation in the C9ORF72 gene is bvFTD. However, various other symptoms may also be observed, such as svPPA, memory deficits, or extrapyramidal motor disorders [120]. From a neuropathological perspective, C9ORF72 cases are characterized by TDP-43 inclusions in both the neocortex and lower motor neurons. Pathology within brain tissue presents primarily as FTLD type B; however, some cases (usually elderly patients without clinical signs of ALS) exhibit features more consistent with a combination of FTLD types A and B [107,120]. Parallel to the TDP-43 pathology, C9ORF72 cases present a unique feature corresponding to NCI and NII lesions immunoreactive for UPS system proteins but not for TDP-43. Using antibodies explicitly directed against DPR allowed us to demonstrate its presence within these TDP-43 negative inclusions. The composition of these lesions reveals that the DPRs resulting from the translation of the antisense transcripts of GGCCCC (GA, GP, and GR) are the ones that are mainly found [107]. This type of lesion is present throughout the neocortex, the limbic system, the cerebellar cortex, the *basal ganglia*, the *thalamus*, and the upper part of the brain stem.

**Figure 8:**
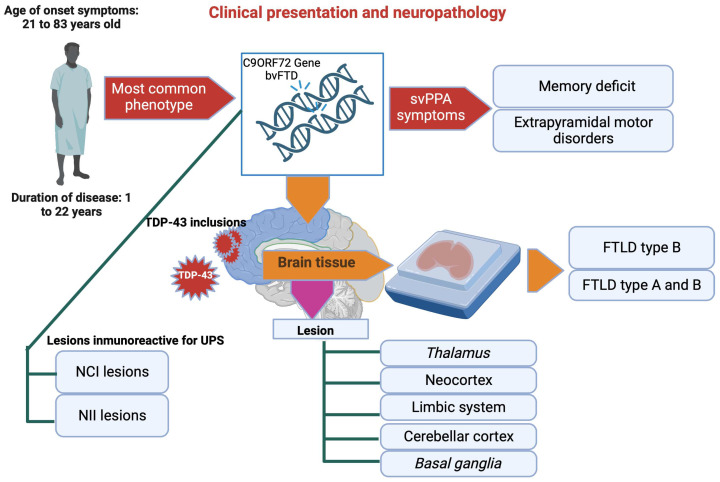
Clinical changes in patients with TDP-43. The alteration of the C9ORF72 gene has an impact on important neurological symptoms, the result of NCI and NII lesions, which affect various brain areas. Created with Biorender.com.

In contrast to the clinical symptoms, DPR pathology distribution is relatively similar among different C9ORF72 cases; there is currently no correlation between DPRs, TDP-43 pathology, and neuronal death [107]. Therefore, the importance of DPR pathology compared to TDP-43 pathology in developing the disease is still debatable. Cases with a C9ORF72 mutation also show intraneuronal RNA foci composed of sense and antisense transcripts of GGCCCC repeats. These foci are present in 50% of neuronal nuclei but rarely in the cytoplasm and glial cells (Figure 8).

## 7. Other Genes Involved

In addition to C9ORF72 and GRN, which represent the primary genes involved in familial cases of FTLD-PDD, several other genes play a role in the development of this pathology. Among them are the genes encoding TDP-43, TARDBP, and VCP.

### 7.1. TARDBP Gene

Most of the mutations that affect the gene that encodes TDP-43 are usually associated with cases of motor neuron disease (MND); however, cognitive disorders (bvFTD and svPPA) have been described in two patients with MND with a mutation in the TARDBP gene [121]. A case has been described as a carrier of the TARDBP K263E mutation who developed FTD, progressive supranuclear palsy (PSP), and chorea; furthermore, these symptoms were correlated with the presence of TDP-43 inclusions in the brainstem and subcortical nuclei, both in neurons and glia. The pathophysiological mechanisms resulting from these TARDBP gene mutations remain to be elucidated. However, it is interesting to note that most of the mutations are located in the C-terminal part of TDP-43, leading to a delocalization of the protein in the cytosolic compartment. Therefore, it is highly likely that these mutations lead to toxic cytosolic gain-of-function and nuclear loss-of-function. From the histopathological point of view, the lesions present a particular profile with a mixture of NCI, NII, and DN at the level of the subcortical regions. At the same time, the cortex shows mainly DN-type lesions [121].

### 7.2. VCP Gene

Mutations in the VCP gene result in a rare syndrome called inclusion body myopathy with early-onset Paget disease and frontotemporal dementia (IBMPFD), affecting skeletal muscle, bone, and the nervous system. Valosin containing protein belongs to the triple-A, ATPase family and plays a role in many cellular processes related to protein homeostasis. In particular, VCP promotes the targeting of ubiquitinated proteins to the proteasome, and the degradation of protein aggregates by autophagy. The VCP gene mutation is also at the origin of an alteration in autophagosome maturation [122]. Patients with VCP mutation present a particular neuropathological signature with many NII-type lesions and DN in the cortex (FTLD-TDP type D) [123]. Other genes would also be associated with developing the TDP-43 pathology; however, their participation is less clear than for the genes mentioned above. Among them are the genes coding for sequestrasome 1, ubiquitin 2, tank-binding kinase 1 (TBK1), or optineurin. The neuropathological data of these genes are currently not sufficient to be able to establish correlations.

## 8. Minority Subclasses of FTDL and Related Syndromes

We have seen that in most cases of FTLD, the tau or the TDP-43 proteins are aggregated; however, in the remaining 5 to 10% of cases, neither of these two proteins is found during neuropathological analysis. In most cases, the FET family proteins (fused in sarcoma, FUS; Ewing sarcoma, EWS; and TATA box binding protein associated factor 2N, TAF15) aggregate within neurons. In rare cases, only proteins linked to the ubiquitin proteasome system (FTLD-UPS) could be identified [124].

## 9. Frontotemporal Lobar Degeneration with FUS (FTLD-FET)

In 2009, mutations in the FUS gene were discovered in familial cases of ALS (ALS-FUS) [123,125]. Because of the confounding clinical signs between ALS and FTLD, but also between FUS and TDP-43, it was hypothesized that FUS aggregates in 5–10% of FTLD cases without tau or TDP-43 lesions. Several studies confirmed this hypothesis thanks to immunological tools specifically directed at FUS. Indeed, in most FTLD cases without tau or TDP-43 pathology, including typical FTLD with ubiquitin inclusions (aFTLD-U) [126], neuronal intermediate filament inclusion disease (NIFID) [127], and basophilic inclusion body disease (BIBD) cases, the presence of inclusions positive for FUS labeling is observed. Subsequently, studies revealed that these lesions were also composed of EWS and TAF15 proteins, as well as transportin 1 (Trn1), which is responsible for targeting FET proteins within the nucleus [128]. Therefore, aFLTD-U, NIFID, BIBD, and FTLD cases characterized by FET protein-positive inclusions were grouped under the term FTLD-FET (for FTLD with FUS, EWS, and TAF15 inclusions). The FET proteins were initially found as part of fusion oncogenes that cause different specific types of cancer in humans. Like TDP-43, these proteins are ubiquitously expressed among the nucleic acid-binding proteins. Therefore, they participate in the metabolism in multiple ways, including regulation of transcription, RNA transport, miRNA maturation, and DNA repair [129]. To do this, they have an RNA recognition domain. FET proteins are primarily localized at the nuclear level but continually move between the nucleus and cytoplasm through nuclear localization and export signals. The physiological functions of FET proteins in the brain are not yet fully understood, but recent studies suggest an essential role in regulating RNA-encoding factors involved in neuronal structure and plasticity, and maintaining dendritic integrity [129]. Like TDP-43, cells displaying FET protein inclusions are characterized by partial or complete loss of these proteins in the nuclear compartment. Several mechanisms have been proposed to explain this delocalization:

1. First, a dysfunction of the Trn1 protein, resulting from a genetic variation of the TNPO1 gene or a post-translational modification, could decrease the efficiency of FET protein transport to the nucleus. However, the absence of other proteins carried by Trn1 within the aggregates, such as heterogeneous nuclear ribonucleoprotein A1 (hnRNPA1), makes this hypothesis less plausible [124].
2. Post-translational modifications, absent under physiological conditions, can also affect the FET proteins. Interestingly, hypomethylation of arginine residues in a region close to the NLS of FET proteins is observed.

**Figure 9:**
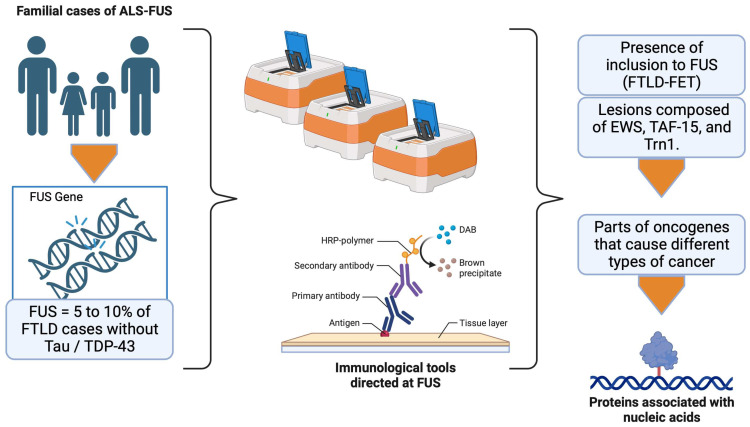
ALS-FUS. The opportune detection of ALS-FUS is carried out with immunological tools, where the objective is the detection of inclusions as well as characteristic lesions. Created with Biorender.com.

This would promote the affinity of the FET proteins for Trn1 and lead to the formation of an inseparable complex, thus favoring the formation of cytoplasmic inclusions [130]. Although the cause of this hypomethylation has yet to be determined, it explains why the aggregates present in FTLD-FETs contain both FET and Trn1 proteins (Figure 9).

## 10. FTLD-FET Subtypes

### 10.1. aFTLD-U

The subtype aFTLD-U corresponds to a group of FTLDs distinguished by the absence of TDP-43 pathology, without clinical symptoms and characteristics of “atypical” pathologies. The onset of bvFTD is generally early in these cases, with a rapid evolution of psychobehavioral disorders without impairment of language and motor pathways.

In addition to frontotemporal atrophy at a macroscopic level, most cases present with degeneration of the anterior part of the *striatum* and sclerosis of the *hippocampus.* Lesions appear NCI-like, particularly small and compact, and are immunoreactive for FET, ubiquitin, and p62 proteins. Regarding their location, they are found mainly in the frontal and temporal neocortex, the *hippocampus*, and the *striatum* [34,124]. Surprisingly, vermiform lesions of NII are also seen within the dentate cortex *gyrus* and pyramidal neurons. Compared with other FTLD-FET cases, aFTLD-U shows less pathology in subcortical regions.

### 10.2. NIFID

NIFID is a rare neurodegenerative disease presenting early FTD symptoms associated with pyramidal and extrapyramidal motor abnormalities [131]. These cases were first distinguished by their unique neuropathologic signature, including different NCI and NII immunoreactive lesions for all intermediate neurofilaments [131]. In addition, NIFID cases present significant FET pathology, including inclusions of variable morphology in many brain regions. The cerebral cortex, *hippocampus*, lower motor neurons, and various subcortical regions, such as the *basal ganglia* and brain stem, are affected. So, NII-like vermiform inclusions, similar to those found in aFLTD-U cases, are also regularly seen, but only in the *hippocampus*, therefore playing a central role in the pathophysiological process in patients with NIFID [126].

### 10.3. BIBD

**Figure 10:**
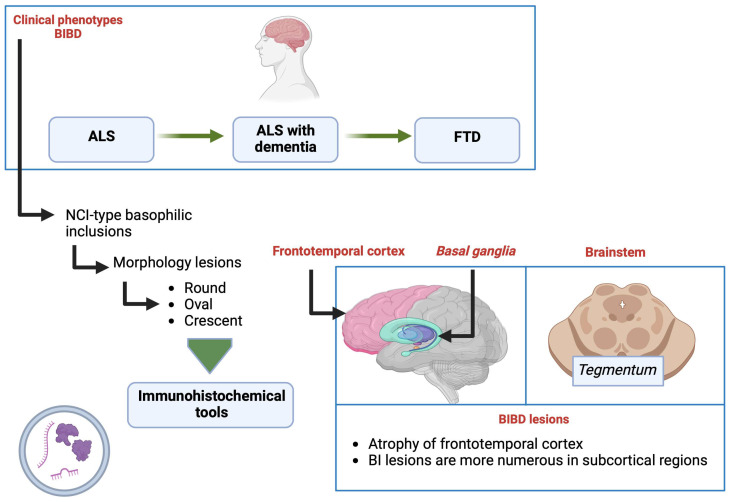
Changes by clinical phenotypes. Lesions are detected by immunohistochemical tools. Mainly BIBD lesions that occur at specific sites in the brain. Created with Biorender.com.

BIBD is also clinically and neuropathologically heterogeneous. Thus, the phenotypes show clinical presentations ranging from ALS to FTD, including ALS with associated dementia. The main neuropathological specificity consists of NCI-type inclusions that can be labeled with primary probes. It appears grey/bluish when stained with hematoxylin and eosin, hence the term basophilic inclusions (BI). The morphology of these lesions is variable (round, oval, or crescent-shaped), and it is possible to detect them using RNA-directed immunohistochemical tools. Although cases with BIBD show atrophy of the frontotemporal cortex, BI lesions are usually more numerous in subcortical regions, such as the *basal ganglia* and brainstem *tegmentum*. In addition to BI lesions, NCIs composed of FET proteins are found in many cortical and subcortical regions, such as NIFID [132]. However, the severity of the pathology in the *hippocampus* and *striatum* is more variable, and NII is not observed (Figure 10).

## 11. Genetics in FTLD-FET

Among the FET proteins, FUS represents the protein most involved in the familial forms: in fact, many nonsense mutations can affect the C-terminal region of FUS and are responsible for approximately 4% of familial forms of ALS. These mutations favor the appearance of the pathology by altering the nuclear localization signal of FUS. The reduction in the interaction between FUS and Trn1 that results from these mutations leads to the formation of FUS inclusions in the cytoplasm [133]. Thus, ALS-FUS cases are characterized by inclusions consisting of FUS but not the other FET proteins [34]. Genetic variations of TAF15 and EWS have also been described in some ALS patients. However, no associated pathology has been described, and there do not seem to be any consequences on the functioning of the proteins [134]. The role of mutations in the genes encoding FET proteins is highly controversial. Most cases of FTLD-FET are sporadic [135]. Although FUS mutations have been reported in some cases with clinical signs of FTD, they have generally been associated with ALS. The analysis of the genes that code for N-arginine-methyltransferases, involved in the methylation of FET proteins, also did not reveal any mutation or alteration in their expression [136].

## 12. FTLD-UPS

Despite the discovery of TDP-43 and FET proteins in most FTLD cases without tau pathology, there are still a small number of cases distinguished only by the presence of inclusions immunoreactive for FTLD-UPS. Among these cases, hereditary cases were associated with a mutation in the gene that encodes a protein involved in forming multivesicular bodies, CHMP2B [88]. The first mutation was initially identified in a subpopulation from Denmark; mutations were subsequently discovered in other families. Patients affected by a CHMP2B mutation present NCI-type granular lesions, mainly in the granular cells of the dentate *gyrus*. These lesions stain positive for ubiquitin and p62 on immunohistochemistry but negative for tau, TDP-43, or FUS. However, it remains to be clarified whether FTLD-UPS results from accumulating an as-yet unidentified protein or a more general lysosomal and endosomal trafficking dysfunction [88].

## 13. Other Pathologies

Several neurodegenerative disorders can be considered FTLD, such as hereditary spheroid leukoencephalopathy or Nasu–Hakola disease [137,138]. In addition, a recent study described an FTD-like syndrome in a family carrying a mutation in the gene encoding the PRKAR1B protein (by a CAMP-dependent protein kinase type-I beta regulatory subunit) [139]. The neuropathological analysis highlights unique NCI lesions consisting of PRKAR1B and the different neurofilaments, suggesting that it could be a rare subtype of NIFID without FUS pathology. Similarly, several other neurodegenerative diseases may present a large part of the diagnostic criteria associated with vcFTD, termed “frontal variants”. Among these variants, AD is the most common and could account for 17% of FTD cases. There are also some FTLD cases in which no lesion has yet been detected, despite using a wide range of immunohistochemical tools; and these cases, formerly called dementia lacking distinctive histopathology (DLDH), are now classified as FTLD-ni (no inclusions) [33].

## 14. LATE

**Figure 11:**
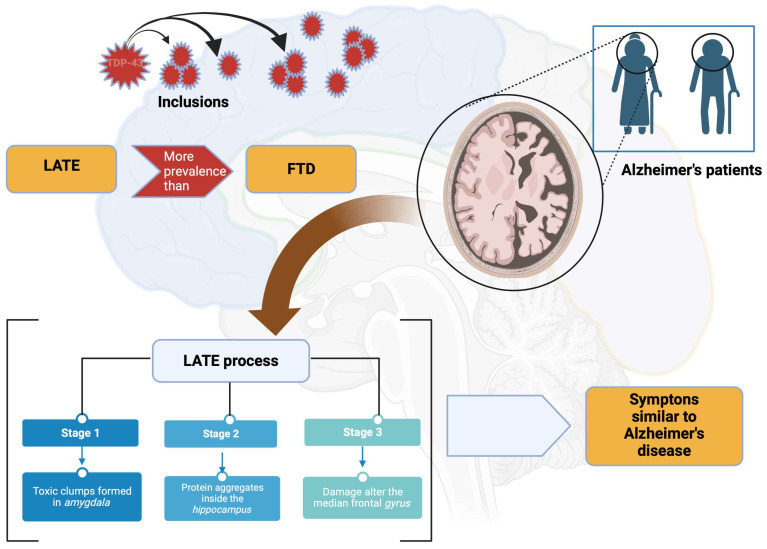
LATE and FTD. It is common for LATE to occur more often than FTD. It should be noted that a large number of elderly Alzheimer’s patients may be manifesting the pathology due to LATE in some of its stages. Created with Biorender.com.

A study has shed light on a new neurocognitive pathology linked to the TDP-43 protein. Discovered by a college of experts led by Professor Peter Nelson of the University of Kentucky, this new disease has been dubbed “TDP-43 predominantly limbic age-related encephalopathy” [8]. According to the international scientific community, this would be one of the most important discoveries related to degenerative diseases in recent decades. According to the researchers behind this description, LATE would be more prevalent than FTD, a condition related to AD [63]. In other words, many patients diagnosed with AD may suffer from TDP-43 age-related predominantly limbic encephalopathy. This new clinical entity is characterized by the presence of the TDP-43 protein outside the nuclei of brain cells where it should usually be found. As part of this neurodegeneration, this protein accumulates abnormally in different regions of the brain, like the tau and beta-amyloid proteins involved in AD [63,107,112] (Figure 11).

This process is usually classified into three stages:

- Stage 1: toxic clumps are formed in the *amygdala*, a region of the brain involved in managing emotions;
- Stage 2: protein aggregates spread inside the *hippocampus*, which plays an essential role in memory processes;
- Stage 3: they reach the median frontal *gyrus* that controls space–time orientation [63,107,112] (Figure 11).

### 14.1. LATE Symptoms

The symptoms are comparable to those of AD. Regarding the latter, the patients present modalities of expression of the pathology that are often very variable and evolutionary [62,63]. Among the main clinical signs of LATE are:

- Memory loss, especially concerning recent events;
- Difficulties in carrying out activities and tasks inherent to daily life;
- Language disorders;
- Loss of motivation;
- Spatiotemporal disorientation;
- Mood swings;
- Behavior problems [63,94].

### 14.2. Causes of LATE

Recently discovered, TDP-43 age-related predominantly limbic encephalopathy is far from having revealed all its secrets. The mechanisms underlying the pathological accumulation of the TDP-43 protein in the different areas of the brain are still unknown. It is known that the appearance of lesions usually occurs in significantly older adults (around 80 years of age). Unlike Alzheimer’s, a disease that occurs at about 65 to 70 years of age, for some unknown reason, the TAR DNA-binding protein TDP-43 begins to malfunction and attaches to nerve cells, causing their death. These mutations would thus be at the origin of irreversible cytoplasmic accumulations. Therefore, for this protein, we seek to understand the mechanisms that underlie these toxic aggregations and develop therapies adapted to the particularities of the clinical signs generated. This toxic aggregate exists in neurodegenerative pathologies such as ALS [106,129].

The warning signs of age-related TDP-43 limbic-predominant encephalopathy can take on different aspects. If many do not show a pathological character at an advanced age, their recurrence and repetition should lead to physician consultation. Early detection and the fastest possible treatment are two essential conditions to stop the progression of the pathology. Unfortunately, this encephalopathy is not yet the subject of a marked diagnostic pathway. Most cases of LATE, when detected, are similar to AD [94,124].

As a new diagnostic entity, LATE remains challenging to differentiate from AD, which shares many commonalities. That is why researchers have estimated that about one third of 85-year-olds diagnosed with the best-known degenerative condition may have TDP-43 age-related predominantly limbic encephalopathy. Physicians currently have very few clinical data to support their diagnostic process, and no known ante-mortem biological biomarkers can reveal the disease with certainty. Therefore, the diagnosis is neuro-pathophysiological and can only be formally confirmed by performing a pathological study of the brain tissue after the patient’s death (a post-mortem diagnosis). But work continues to establish objective diagnostic criteria based on formal medical examinations [62,63].

Medical neuroimaging seems to have a promising future since recent studies have shown that people affected by this particular encephalopathy will present atrophy in the medial temporal lobes and frontal cortex, as well as a particularly damaged *amygdala* and *hippocampus* [63].

Thus, regarding the differences between FTLD and LATE, the first corresponds to several anatomopathological entities that have as a common consequence frontal and anterior temporal degeneration. Behavioral FTLD typically has dysexecutive disorders at the forefront with relative preservation of memory and visuospatial domains, while the second is characterized by an isolated amnestic syndrome, which progresses slowly in the absence of other comorbidities, and more rapidly in the presence of Alzheimer’s disease (AD) or other dementia pathology, specially FTLD. In the absence of specific markers, the combination of imaging data (MRI, FTDP-TDP) and plasma markers may contribute to the differential diagnosis [74].

## 15. Conclusions

The precise pathophysiological mechanisms of frontotemporal-TDP and limbic age-related encephalopathy are not fully understood. Nevertheless, age-related changes in the brain, including protein misfolding and neuroinflammation, are considered contributing factors. These disorders have a complex interplay of genetic and environmental factors in their development and progression. Familial cases of frontotemporal-TDP indicate the involvement of specific gene mutations, while sporadic cases point to potential genetic predisposition and other unidentified triggers. Understanding the pathophysiological and genetic basis for both disorders is crucial for early diagnosis, targeted therapies, and the development of disease-modifying treatments in the future. Further research into these neurocognitive disorders holds promise for enhancing the quality of life for affected individuals and their families.

## Figures and Tables

**Table 1:** Summary of mutations associated with FLTD-TDP.

Mutation	Gene	Protein	Reference
C9ORF72 expansion	C9ORF72	TDP-43	[75,76,77]
GRN mutation	GRN	Progranulin	[78,79,80]
MAPT mutation	MAPT	Tau	[81,82]
TBK1 mutation	TBK1	TANK-binding kinase 1	[83]
VCP mutation	VCP	Valosin-containing protein	[84,85,86]
CHMP2B mutation	CHMP2B	Charged multivesicular body protein 2B	[87,88]
SQSTM1 mutation	SQSTM1	Sequestosome-1 (p62)	[89,90]
TIA1 mutation	TIA1	TIA-1 RNA-binding protein	[91]

## Data Availability

Data are available on request from the authors.

## References

[1] Ou S.H., Wu F., Harrich D., Garcia-Martinez L.F., Gaynor R.B. (1995). Cloning and characterization of a novel cellular protein, TDP-43, that binds to human immunodeficiency virus type 1 TAR DNA sequence motifs. J. Virol..

[2] Fuentealba R.A., Udan M., Bell S., Wegorzewska I., Shao J., Diamond M.I., Weihl C.C., Baloh R.H. (2010). Interaction with polyglutamine aggregates reveals a Q/N-rich domain in TDP-43. J. Biol. Chem..

[3] Buratti E., Baralle F.E. (2001). Characterization and functional implications of the RNA binding properties of nuclear factor TDP-43, a novel splicing regulator of CFTR exon 9. J. Biol. Chem..

[4] Sephton C.F., Cenik C., Kucukural A., Dammer E.B., Cenik B., Han Y., Dewey C.M., Roth F.P., Herz J., Peng J. (2011). Identification of neuronal RNA targets of TDP-43-containing ribonucleoprotein complexes. J. Biol. Chem..

[5] Polymenidou M., Lagier-Tourenne C., Hutt K.R., Huelga S.C., Moran J., Liang T.Y., Ling S.C., Sun E., Wancewicz E., Mazur C. (2011). Long pre-mRNA depletion and RNA missplicing contribute to neuronal vulnerability from loss of TDP-43. Nat. Neurosci..

[6] Tollervey J.R., Curk T., Rogelj B., Briese M., Cereda M., Kayikci M., Konig J., Hortobagyi T., Nishimura A.L., Zupunski V. (2011). Characterizing the RNA targets and position-dependent splicing regulation by TDP-43. Nat. Neurosci..

[7] Ayala Y.M., De Conti L., Avendaño-Vázquez S.E., Dhir A., Romano M., D’Ambrogio A., Tollervey J., Ule J., Baralle M., Buratti E. (2011). TDP-43 regulates its mRNA levels through a negative feedback loop. EMBO J..

[8] Nelson P.T., Keller J.N. (2007). RNA in brain disease: No longer just “the messenger in the middle”. J. Neuropathol. Exp. Neurol..

[9] Fire A., Xu S., Montgomery M.K., Kostas S.A., Driver S.E., Mello C.C. (1998). Potent and specific genetic interference by double-stranded RNA in Caenorhabditis elegans. Nature.

[10] Lee Y., Ahn C., Han J., Choi H., Kim J., Yim J., Lee J., Provost P., Radmark O., Kim S. (2003). The nuclear RNase III Drosha initiates microRNA processing. Nature.

[11] Cullen B.R. (2004). Derivation and function of small interfering RNAs and microRNAs. Virus Res..

[12] Casafont I., Bengoechea R., Tapia O., Berciano M.T., Lafarga M. (2009). TDP-43 localizes in mRNA transcription and processing sites in mammalian neurons. J. Struct. Biol..

[13] Colombrita C., Onesto E., Buratti E., de la Grange P., Gumina V., Baralle F.E., Silani V., Ratti A. (2015). From transcriptomic to protein level changes in TDP-43 and FUS loss-of-function cell models. Biochim. Biophys. Acta.

[14] Roberts T.C., Morris K.V., Wood M.J.A. (2014). The role of long non-coding RNAs in neurodevelopment, brain function and neurological disease. Philos. Trans. R. Soc. B Biol. Sci..

[15] Nishimoto Y., Nakagawa S., Hirose T., Okano H.J., Takao M., Shibata S., Suyama S., Kuwako K., Imai T., Murayama S. (2013). The long non-coding RNA nuclear-enriched abundant transcript 1_2 induces paraspeckle formation in the motor neuron during the early phase of amyotrophic lateral sclerosis. Mol. Brain.

[16] Morimoto M., Boerkoel C.F. (2013). The role of nuclear bodies in gene expression and disease. Biology.

[17] Lourenco G.F., Janitz M., Huang Y., Halliday G.M. (2015). Long noncoding RNAs in TDP-43 and FUS/TLS-related frontotemporal lobar degeneration (FTLD). Neurobiol. Dis..

[18] Fiesel F.C., Schurr C., Weber S.S., Kahle P.J. (2011). TDP-43 knockdown impairs neurite outgrowth dependent on its target histone deacetylase 6. Mol. Neurodegener..

[19] Costessi L., Porro F., Iaconcig A., Muro A.F. (2014). TDP-43 regulates β-adducin (Add2) transcript stability. RNA Biol..

[20] Colombrita C., Onesto E., Megiorni F., Pizzuti A., Baralle F.E., Buratti E., Silani V., Ratti A. (2012). TDP-43 and FUS RNA-binding proteins bind distinct sets of cytoplasmic messenger RNAs and differently regulate their post-transcriptional fate in motoneuron-like cells. J. Biol. Chem..

[21] Lee S., Lee T.A., Lee E., Kang S., Park A., Kim S.W., Park H.J., Yoon J.H., Ha S.J., Park T. (2015). Identification of a subnuclear body involved in sequence-specific cytokine RNA processing. Nat. Commun..

[22] Liu-Yesucevitz L., Bassell G.J., Gitler A.D., Hart A.C., Klann E., Richter J.D., Warren S.T., Wolozin B. (2011). Local RNA translation at the synapse and in disease. J. Neurosci..

[23] Fallini C., Bassell G.J., Rossoll W. (2012). The ALS disease protein TDP-43 is actively transported in motor neuron axons and regulates axon outgrowth. Hum. Mol. Genet..

[24] Alami N.H., Smith R.B., Carrasco M.A., Williams L.A., Winborn C.S., Han S.S.W., Kiskinis E., Winborn B., Freibaum B.D., Kanagaraj A. (2014). Axonal transport of TDP-43 mRNA granules is impaired by ALS-causing mutations. Neuron.

[25] Wang Y.F., Sun M.Y., Hou Q., Parpura V. (2013). Hyposmolality differentially and spatiotemporally modulates levels of glutamine synthetase and serine racemase in rat supraoptic nucleus. Glia.

[26] Freibaum B.D., Chitta R.K., High A.A., Taylor J.P. (2010). Global analysis of TDP-43 interacting proteins reveals strong association with RNA splicing and translation machinery. J. Proteome Res..

[27] Higashi S., Kabuta T., Nagai Y., Tsuchiya Y., Akiyama H., Wada K. (2013). TDP-43 associates with stalled ribosomes and contributes to cell survival during cellular stress. J. Neurochem..

[28] Coyne A.N., Siddegowda B.B., Estes P.S., Johannesmeyer J., Kovalik T., Daniel S.G., Pearson A., Bowser R., Zarnescu D.C. (2014). Futsch/MAP1B mRNA is a translational target of TDP-43 and is neuroprotective in a Drosophila model of amyotrophic lateral sclerosis. J. Neurosci..

[29] Majumder P., Chen Y.T., Bose J.K., Wu C.C., Cheng W.C., Cheng S.J., Fang Y.H., Chen Y.L., Tsai K.J., Lien C.C. (2012). TDP-43 regulates the mammalian spinogenesis through translational repression of Rac1. Acta Neuropathol..

[30] MacNair L., Xiao S., Miletic D., Ghani M., Julien J.P., Keith J., Zinman L., Rogaeva E., Robertson J. (2016). MTHFSD and DDX58 are novel RNA-binding proteins abnormally regulated in amyotrophic lateral sclerosis. Brain.

[31] Lee E.B., Lee V.M., Trojanowski J.Q. (2011). Gains or losses: Molecular mechanisms of TDP43-mediated neurodegeneration. Nat. Rev. Neurosci..

[32] Mackenzie I.R., Neumann M. (2016). Molecular neuropathology of frontotemporal dementia: Insights into disease mechanisms from post-mortem studies. J. Neurochem..

[33] Josephs K.A., Stroh A., Dugger B., Dickson D.W. (2009). Evaluation of subcortical pathology and clinical correlations in FTLD-U subtypes. Acta Neuropathol..

[34] Mackenzie I.R., Neumann M., Baborie A., Sampathu D.M., Du Plessis D., Jaros E., Perry R.H., Trojanowski J.Q., Mann D.M., Lee V.M. (2011). A harmonized classification system for FTLD-TDP pathology. Acta Neuropathol..

[35] Hasegawa M., Arai T., Nonaka T., Kametani F., Yoshida M., Hashizume Y., Beach T.G., Buratti E., Baralle F., Morita M. (2008). Phosphorylated TDP-43 in frontotemporal lobar degeneration and amyotrophic lateral sclerosis. Ann. Neurol..

[36] Neumann M., Kwong L.K., Lee E.B., Kremmer E., Flatley A., Xu Y., Forman M.S., Troost D., Kretzschmar H.A., Trojanowski J.Q. (2009). Phosphorylation of S409/410 of TDP-43 is a consistent feature in all sporadic and familial forms of TDP-43 proteinopathies. Acta Neuropathol..

[37] Strong M.J., Volkening K., Hammond R., Yang W., Strong W., Leystra-Lantz C., Shoesmith C. (2007). TDP43 is a human low molecular weight neurofilament (hNFL) mRNA-binding protein. Mol. Cell. Neurosci..

[38] Kametani F., Nonaka T., Suzuki T., Arai T., Dohmae N., Akiyama H., Hasegawa M. (2009). Identification of casein kinase-1 phosphorylation sites on TDP-43. Biochem. Biophys. Res. Commun..

[39] Igaz L.M., Kwong L.K., Chen-Plotkin A., Winton M.J., Unger T.L., Xu Y., Neumann M., Trojanowski J.Q., Lee V.M. (2009). Expression of TDP-43 C-terminal Fragments in Vitro Recapitulates Pathological Features of TDP-43 Proteinopathies. J. Biol. Chem..

[40] Nonaka T., Kametani F., Arai T., Akiyama H., Hasegawa M. (2009). Truncation and pathogenic mutations facilitate the formation of intracellular aggregates of TDP-43. Hum. Mol. Genet..

[41] Winton M.J., Igaz L.M., Wong M.M., Kwong L.K., Trojanowski J.Q., Lee V.M. (2008). Disturbance of nuclear and cytoplasmic TAR DNA-binding protein (TDP-43) induces disease-like redistribution, sequestration, and aggregate formation. J. Biol. Chem..

[42] Wegorzewska I., Bell S., Cairns N.J., Miller T.M., Baloh R.H. (2009). TDP-43 mutant transgenic mice develop features of ALS and frontotemporal lobar degeneration. Proc. Natl. Acad. Sci. USA.

[43] Xu Y.F., Gendron T.F., Zhang Y.J., Lin W.L., D’Alton S., Sheng H., Casey M.C., Tong J., Knight J., Yu X. (2010). Wild-type human TDP-43 expression causes TDP-43 phosphorylation, mitochondrial aggregation, motor deficits, and early mortality in transgenic mice. J. Neurosci..

[44] Zhang Y.J., Gendron T.F., Xu Y.F., Ko L.W., Yen S.H., Petrucelli L. (2010). Phosphorylation regulates proteasomal-mediated degradation and solubility of TAR DNA binding protein-43 C-terminal fragments. Mol. Neurodegener..

[45] Liachko N.F., McMillan P.J., Guthrie C.R., Bird T.D., Leverenz J.B., Kraemer B.C. (2013). CDC7 inhibition blocks pathological TDP-43 phosphorylation and neurodegeneration. Ann. Neurol..

[46] Liachko N.F., McMillan P.J., Strovas T.J., Loomis E., Greenup L., Murrell J.R., Ghetti B., Raskind M.A., Montine T.J., Bird T.D. (2014). The tau tubulin kinases TTBK1/2 promote accumulation of pathological TDP-43. PLoS Genet..

[47] Li H.Y., Yeh P.A., Chiu H.C., Tang C.Y., Tu B.P. (2011). Hyperphosphorylation as a defense mechanism to reduce TDP-43 aggregation. PLoS ONE.

[48] Dormann D., Capell A., Carlson A.M., Shankaran S.S., Rodde R., Neumann M., Kremmer E., Matsuwaki T., Yamanouchi K., Nishihara M. (2009). Proteolytic processing of TAR DNA binding protein-43 by caspases produces C-terminal fragments with disease defining properties independent of progranulin. J. Neurochem..

[49] Iguchi Y., Katsuno M., Takagi S., Ishigaki S., Niwa J., Hasegawa M., Tanaka F., Sobue G. (2012). Oxidative stress induced by glutathione depletion reproduces pathological modifications of TDP-43 linked to TDP-43 proteinopathies. Neurobiol. Dis..

[50] Hebron M.L., Lonskaya I., Sharpe K., Weerasinghe P.P., Algarzae N.K., Shekoyan A.R., Moussa C.E. (2013). Parkin ubiquitinates Tar-DNA binding protein-43 (TDP-43) and promotes its cytosolic accumulation via interaction with histone deacetylase 6 (HDAC6). J. Biol. Chem..

[51] Pesiridis G.S., Tripathy K., Tanik S., Trojanowski J.Q., Lee V.M. (2011). A “two-hit” hypothesis for inclusion formation by carboxyl-terminal fragments of TDP-43 protein linked to RNA depletion and impaired microtubule-dependent transport. J. Biol. Chem..

[52] Low P. (2011). The role of ubiquitin-proteasome system in ageing. Gen. Comp. Endocrinol..

[53] Hans F., Fiesel F.C., Strong J.C., Jäckel S., Rasse T.M., Geisler S., Springer W., Schulz J.B., Voigt A., Kahle P.J. (2014). UBE2E ubiquitin-conjugating enzymes and ubiquitin isopeptidase Y regulate TDP-43 protein ubiquitination. J. Biol. Chem..

[54] Lee B.H., Lee M.J., Park S., Oh D.C., Elsasser S., Chen P.C., Gartner C., Dimova N., Hanna J., Gygi S.P. (2010). Enhancement of proteasome activity by a small-molecule inhibitor of USP14. Nature.

[55] Sreedharan J., Blair I.P., Tripathi V.B., Hu X., Vance C., Rogelj B., Ackerley S., Durnall J.C., Williams K.L., Buratti E. (2008). TDP-43 mutations in familial and sporadic amyotrophic lateral sclerosis. Science.

[56] Zhang Y.J., Xu Y.F., Dickey C.A., Buratti E., Baralle F., Bailey R., Pickering-Brown S., Dickson D., Petrucelli L. (2007). Progranulin mediates caspase-dependent cleavage of TAR DNA binding protein-43. J. Neurosci..

[57] Suzuki H., Lee K., Matsuoka M. (2011). TDP-43-induced death is associated with altered regulation of BIM and Bcl-xL and attenuated by caspase-mediated TDP-43 cleavage. J. Biol. Chem..

[58] Voigt A., Herholz D., Fiesel F.C., Kaur K., Müller D., Karsten P., Weber S.S., Kahle P.J., Marquardt T., Schulz J.B. (2010). TDP-43-mediated neuron loss in vivo requires RNA-binding activity. PLoS ONE.

[59] Colombrita C., Zennaro E., Fallini C., Weber M., Sommacal A., Buratti E., Silani V., Ratti A. (2009). TDP-43 is recruited to stress granules in conditions of oxidative insult. J. Neurochem..

[60] Yamashita M., Nonaka T., Hirai S., Miwa A., Okado H., Arai T., Hosokawa M., Akiyama H., Hasegawa M. (2014). Distinct pathways leading to TDP-43-induced cellular dysfunctions. Hum. Mol. Genet..

[61] Igaz L.M., Kwong L.K., Lee E.B., Chen-Plotkin A., Swanson E., Unger T., Malunda J., Xu Y., Winton M.J., Trojanowski J.Q. (2011). Dysregulation of the ALS-associated gene TDP-43 leads to neuronal death and degeneration in mice. J. Clin. Investig..

[62] Fang Y.S., Tsai K.J., Chang Y.J., Kao P., Woods R., Kuo P.H., Wu C.C., Liao J.Y., Chou S.C., Lin V. (2014). Full-length TDP-43 forms toxic amyloid oligomers that are present in frontotemporal lobar dementia-TDP patients. Nat. Commun..

[63] Brettschneider J., Del Tredici K., Irwin D.J., Grossman M., Robinson J.L., Toledo J.B., Fang L., Van Deerlin V.M., Ludolph A.C., Lee V.M. (2014). Sequential distribution of pTDP-43 pathology in behavioral variant frontotemporal dementia (bvFTD). Acta Neuropathol..

[64] Furukawa Y., Kaneko K., Nukina N. (2011). Molecular properties of TAR DNA binding protein-43 fragments are dependent upon its cleavage site. Biochim. Biophys. Acta.

[65] Kleinberger G., Capell A., Haass C., Van Broeckhoven C. (2013). Mechanisms of granulin deficiency: Lessons from cellular and animal models. Mol. Neurobiol..

[66] Bateman A., Belcourt D., Bennett H., Lazure C., Solomon S. (1990). Granulins, a novel class of peptide from leukocytes. Biochem. Biophys. Res. Commun..

[67] Ahmed Z., Sheng H., Xu Y.F., Lin W.L., Innes A.E., Gass J., Yu X., Hou H., Chiba S., Yamanouchi K. (2010). Accelerated lipofuscinosis and ubiquitination in granulin knockout mice suggest a role for progranulin in successful aging. Am. J. Pathol..

[68] Naphade S.B., Kigerl K.A., Jakeman L.B., Kostyk S.K., Popovich P.G., Kuret J. (2010). Progranulin expression is upregulated after spinal contusion in mice. Acta Neuropathol..

[69] Philips T., De Muynck L., Thu H.N., Weynants B., Vanacker P., Dhondt J., Sleegers K., Schelhaas H.J., Verbeek M., Vandenberghe R. (2010). Microglial upregulation of progranulin as a marker of motor neuron degeneration. J. Neuropathol. Exp. Neurol..

[70] Finch N., Carrasquillo M.M., Baker M., Rutherford N.J., Coppola G., Dejesus-Hernandez M., Crook R., Hunter T., Ghidoni R., Benussi L. (2011). TMEM106B regulates progranulin levels and the penetrance of FTLD in GRN mutation carriers. Neurology.

[71] Cruchaga C., Graff C., Chiang H.H., Wang J., Hinrichs A.L., Spiegel N., Bertelsen S., Mayo K., Norton J.B., Morris J.C. (2011). Association of TMEM106B gene polymorphism with age at onset in granulin mutation carriers and plasma granulin protein levels. Arch Neurol..

[72] Lang C.M., Fellerer K., Schwenk B.M., Kuhn P.H., Kremmer E., Edbauer D., Capell A., Haass C. (2012). Membrane orientation and subcellular localization of transmembrane protein 106B (TMEM106B), a major risk factor for frontotemporal lobar degeneration. J. Biol. Chem..

[73] Chen-Plotkin A.S., Unger T.L., Gallagher M.D., Bill E., Kwong L.K., Volpicelli-Daley L., Busch J.I., Akle S., Grossman M., Van Deerlin V. (2012). TMEM106B, the risk gene for frontotemporal dementia, is regulated by the microRNA-132/212 cluster and affects progranulin pathways. J. Neurosci..

[74] Dubois B., Michon A., Léger J.M., Mas J.L. (2015). Démences.

[75] Sonobe Y., Lee S., Krishnan G., Gu Y., Kwon D.Y., Gao F.B., Roos R.P., Kratsios P. (2023). Translation of dipeptide repeat proteins in C9ORF72 ALS/FTD through unique and redundant AUG initiation codons. eLife.

[76] Kortazar-Zubizarreta I., Manero-Azua A., Afonso-Agüera J., de Nanclares G.P. (2023). C9ORF72 Gene GGGGCC Hexanucleotide Expansion: A High Clinical Variability from Amyotrophic Lateral Sclerosis to Frontotemporal Dementia. J. Pers. Med..

[77] Črnigoj M.M., Čerček U., Yin X., Ho M.T., Lampret B.R., Neumann M., Hermann A., Rouleau G., Suter B., Mayr M. (2023). Phenylalanine-tRNA aminoacylation is compromised by ALS/FTD-associated C9orf72 C4G2 repeat RNA. Nat. Commun..

[78] Snowden J.S., Pickering-Brown S.M., Mackenzie I.R., Richardson A.M., Varma A., Neary D., Mann D.M. (2006). Progranulin gene mutations associated with frontotemporal dementia and progressive non-fluent aphasia. Brain.

[79] Saracino D., Ferrieux S., Noguès-Lassiaille M., Houot M., Funkiewiez A., Sellami L., Deramecourt V., Pasquier F., Couratier P., Pariente J. (2021). Primary Progressive Aphasia Associated With GRN Mutations: New Insights Into the Nonamyloid Logopenic Variant. Neurology.

[80] Puoti G., Lerza M.C., Ferretti M.G., Bugiani O., Tagliavini F., Rossi G. (2014). A mutation in the 5’-UTR of GRN gene associated with frontotemporal lobar degeneration: Phenotypic variability and possible pathogenetic mechanisms. J. Alzheimers Dis..

[81] Hutton M. (2001). Missense and splice site mutations in tau associated with FTDP-17: Multiple pathogenic mechanisms. Neurology.

[82] Hutton M., Lendon C.L., Rizzu P., Baker M., Froelich S., Houlden H., Pickering-Brown S., Chakraverty S., Isaacs A., Grover A. (1998). Association of missense and 5’-splice-site mutations in tau with the inherited dementia FTDP-17. Nature.

[83] Pottier C., Ravenscroft T.A., Brown P.H., Finch N.A., Baker M., Parsons M., Asmann Y.W., Ren Y., Christopher E., Levitch D. (2016). TYROBP genetic variants in early-onset Alzheimer’s disease. Neurobiol. Aging.

[84] Watts G.D., Wymer J., Kovach M.J., Mehta S.G., Mumm S., Darvish D., Pestronk A., Whyte M.P., Kimonis V.E. (2004). Inclusion body myopathy associated with Paget disease of bone and frontotemporal dementia is caused by mutant valosin-containing protein. Nat. Genet..

[85] Vesa J., Su H., Watts G.D., Krause S., Walter M.C., Martin B., Smith C., Wallace D.C., Kimonis V.E. (2009). Valosin containing protein associated inclusion body myopathy: Abnormal vacuolization, autophagy and cell fusion in myoblasts. Neuromuscul. Disord..

[86] Custer S.K., Neumann M., Lu H., Wright A.C., Taylor J.P. (2010). Transgenic mice expressing mutant forms VCP/p97 recapitulate the full spectrum of IBMPFD including degeneration in muscle, brain and bone. Hum. Mol. Genet..

[87] Li C., Wen Y., Zhao M., Wang Y., Li P., Wang L., Wang S. (2023). A novel splice-site mutation in CHMP2B associated with frontotemporal dementia: The first report from China and literature review. Mol. Genet. Genom. Med..

[88] Skibinski G., Parkinson N.J., Brown J.M., Chakrabarti L., Lloyd S.L., Hummerich H., Nielsen J.E., Hodges J.R., Spillantini M.G., Thusgaard T. (2005). Mutations in the endosomal ESCRTIII-complex subunit CHMP2B in frontotemporal dementia. Nat. Genet..

[89] Rubino E., Rainero I., Chiò A., Rogaeva E., Galimberti D., Fenoglio P., Grinberg Y., Isaia G., Calvo A., Gentile S. (2012). SQSTM1 mutations in frontotemporal lobar degeneration and amyotrophic lateral sclerosis. Neurology.

[90] Lee S., Jeon Y.M., Cha S.J., Kim S., Kwon Y., Jo M., Jang Y.N., Lee S., Kim J., Kim S.R. (2020). PTK2/FAK regulates UPS impairment via SQSTM1/p62 phosphorylation in TARDBP/TDP-43 proteinopathies. Autophagy.

[91] Mackenzie I.R., Nicholson A.M., Sarkar M., Messing J., Purice M.D., Pottier C., Annu K., Baker M., Perkerson R.B., Kurti A. (2017). TIA1 Mutations in Amyotrophic Lateral Sclerosis and Frontotemporal Dementia Promote Phase Separation and Alter Stress Granule Dynamics. Neuron.

[92] Van Damme P., Van Hoecke A., Lambrechts D., Vanacker P., Bogaert E., van Swieten J., Carmeliet P., Van Den Bosch L., Robberecht W. (2008). Progranulin functions as a neurotrophic factor to regulate neurite outgrowth and enhance neuronal survival. J. Cell Biol..

[93] Tapia L., Milnerwood A., Guo A., Mills F., Yoshida E., Vasuta C., Mackenzie I.R., Raymond L., Cynader M., Jia W. (2011). Progranulin deficiency decreases gross neural connectivity but enhances transmission at individual synapses. J. Neurosci..

[94] Kocerha J., Kouri N., Baker M., Finch N., DeJesus-Hernandez M., Gonzalez J., Chidamparam K., Josephs K.A., Boeve B.F., Graff-Radford N.R. (2011). Altered microRNA expression in frontotemporal lobar degeneration with TDP-43 pathology caused by progranulin mutations. BMC Genom..

[95] Lui H., Zhang J., Makinson S.R., Cahill M.K., Kelley K.W., Huang H.Y., Shang Y., Oldham M.C., Martens L.H., Gao F. (2016). Progranulin Deficiency Promotes Circuit-Specific Synaptic Pruning by Microglia via Complement Activation. Cell.

[96] Gass J., Lee W.C., Cook C., Finch N., Stetler C., Jansen-West K., Lewis J., Link C.D., Rademakers R., Nykjaer A. (2012). Progranulin regulates neuronal outgrowth independent of sortilin. Mol. Neurodegener..

[97] Gass J., Prudencio M., Stetler C., Petrucelli L. (2012). Progranulin: An emerging target for FTLD therapies. Brain Res..

[98] Hu F., Padukkavidana T., Vaegter C.B., Brady O.A., Zheng Y., Mackenzie I.R., Feldman H.H., Nykjaer A., Strittmatter S.M. (2010). Sortilin-mediated endocytosis determines levels of the frontotemporal dementia protein, progranulin. Neuron.

[99] Martens L.H., Zhang J., Barmada S.J., Zhou P., Kamiya S., Sun B., Min S.W., Gan L., Finkbeiner S., Huang E.J. (2022). Progranulin deficiency promotes neuroinflammation and neuron loss following toxin-induced injury. J. Clin. Investig..

[100] Suh H.S., Choi N., Tarassishin L., Lee S.C. (2012). Regulation of progranulin expression in human microglia and proteolysis of progranulin by matrix metalloproteinase-12 (MMP-12). PLoS ONE.

[101] Guo A., Tapia L., Bamji S.X., Cynader M.S., Jia W. (2010). Progranulin deficiency leads to enhanced cell vulnerability and TDP-43 translocation in primary neuronal cultures. Brain Res..

[102] Kleinberger G., Wils H., Ponsaerts P., Joris G., Timmermans J.P., Van Broeckhoven C., Kumar-Singh S. (2010). Increased caspase activation and decreased TDP-43 solubility in progranulin knockout cortical cultures. J. Neurochem..

[103] Shankaran S.S., Capell A., Hruscha A.T., Fellerer K., Neumann M., Schmid B., Haass C. (2008). Missense mutations in the progranulin gene linked to frontotemporal lobar degeneration with ubiquitin-immunoreactive inclusions reduce progranulin production and secretion. J. Biol. Chem..

[104] Wils H., Kleinberger G., Pereson S., Janssens J., Capell A., Van Dam D., Cuijt I., Joris G., De Deyn P.P., Haass C. (2012). Cellular ageing, increased mortality and FTLD-TDP-associated neuropathology in progranulin knockout mice. J. Pathol..

[105] Yin F., Dumont M., Banerjee R., Ma Y., Li H., Lin M.T., Beal M.F., Nathan C., Thomas B., Ding A. (2010). Behavioral deficits and progressive neuropathology in progranulin-deficient mice: A mouse model of frontotemporal dementia. FASEB J..

[106] Salazar D.A., Butler V.J., Argouarch A.R., Hsu T.Y., Mason A., Nakamura A., McCurdy H., Cox D., Ng R., Pan G. (2015). The Progranulin Cleavage Products, Granulins, Exacerbate TDP-43 Toxicity and Increase TDP-43 Levels. J. Neurosci..

[107] Mackenzie I.R. (2007). The neuropathology and clinical phenotype of FTD with progranulin mutations. Acta Neuropathol..

[108] Whitwell J.L., Weigand S.D., Boeve B.F., Senjem M.L., Gunter J.L., DeJesus-Hernandez M., Rutherford N.J., Baker M., Knopman D.S., Wszolek Z.K. (2012). Neuroimaging signatures of frontotemporal dementia genetics: C9ORF72, Tau, progranulin and sporadics. Brain.

[109] Sieben A., Van Langenhove T., Engelborghs S., Martin J.J., Boon P., Cras P., De Deyn P.P., Santens P., Van Broeckhoven C., Cruts M. (2012). The genetics and neuropathology of frontotemporal lobar degeneration. Acta Neuropathol..

[110] Hatanpaa K.J., Bigio E.H., Cairns N.J., Womack K.B., Weintraub S., Morris J.C., Foong C., Xiao G., Hladik C., Mantanona T.Y. (2008). TAR DNA-binding protein 43 immunohistochemistry reveals extensive neuritic pathology in FTLD-U: A midwest-southwest consortium for FTLD study. J. Neuropathol. Exp. Neurol..

[111] Josephs K.A., Ahmed Z., Katsuse O., Parisi J.F., Boeve B.F., Knopman D.S., Petersen R.C., Davies P., Duara R., Graff-Radford N.R. (2007). Neuropathologic features of frontotemporal lobar degeneration with ubiquitin-positive inclusions with progranulin gene (PGRN) mutations. J. Neuropathol. Exp. Neurol..

[112] DeJesus-Hernandez M., Mackenzie I.R., Boeve B.F., Boxer A.L., Baker M., Rutherford N.J., Nicholson A.M., Finch N.A., Flynn H., Adamson J. (2011). Expanded GGGGCC hexanucleotide repeat in noncoding region of C9ORF72 causes chromosome 9p-linked FTD and ALS. Neuron.

[113] Woollacott I.O., Mead S. (2014). The C9ORF72 expansion mutation: Gene structure, phenotypic and diagnostic issues. Acta Neuropathol..

[114] Majounie E., Renton A.E., Mok K., Dopper E.G., Waite A., Rollinson S., Chio A., Restagno G., Nicolaou N., Simon-Sanchez J. (2012). Frequency of the C9orf72 hexanucleotide repeat expansion in patients with amyotrophic lateral sclerosis and frontotemporal dementia: A cross-sectional study. Lancet Neurol..

[115] Gendron T.F., Cosio D.M., Petrucelli L. (2013). c9RAN translation: A potential therapeutic target for the treatment of amyotrophic lateral sclerosis and frontotemporal dementia. Expert Opin. Ther. Targets.

[116] Mori K., Weng S.M., Arzberger T., May S., Rentzsch K., Kremmer E., Schmid B., Kretzschmar H.A., Cruts M., Van Broeckhoven C. (2013). The C9orf72 GGGGCC repeat is translated into aggregating dipeptide-repeat proteins in FTLD/ALS. Science.

[117] Caillet-Boudin M.L., Fernandez-Gomez F.J., Tran H., Dhaenens C.M., Buee L., Sergeant N. (2014). Brain pathology in myotonic dystrophy: When tauopathy meets spliceopathy and RNAopathy. Front. Mol. Neurosci..

[118] Zhang Y.J., Gendron T.F., Grima J.C., Sasaguri H., Jansen-West K., Xu Y.F., Katzman R.B., Gass J., Murray M.E., Shinohara M. (2016). C9ORF72 poly(GA) aggregates sequester and impair HR23 and nucleocytoplasmic transport proteins. Nat. Neurosci..

[119] Gallagher M.D., Suh E., Grossman M., Elman L., McCluskey L., Van Swieten J.C., Al-Sarraj S., Neumann M., Gelpi E., Ghetti B. (2014). TMEM106B is a genetic modifier of frontotemporal lobar degeneration with C9orf72 hexanucleotide repeat expansions. Acta Neuropathol..

[120] Hsiung G.Y., DeJesus-Hernandez M., Feldman H.H., Sengdy P., Bouchard-Kerr P., Dwosh E., Butler R., Leung B., Fok A., Rutherford N.J. (2012). Clinical and pathological features of familial frontotemporal dementia caused by C9ORF72 mutation on chromosome 9p. Brain.

[121] Benajiba L., Le Ber I., Camuzat A., Lacoste M., Thomas-Anterion C., Couratier P., Legallic S., Salachas F., Hannequin D., Decousus M. (2009). TARDBP mutations in motoneuron disease with frontotemporal lobar degeneration. Ann. Neurol..

[122] Ju J.S., Weihl C.C. (2010). Inclusion body myopathy, Paget’s disease of the bone and fronto-temporal dementia: A disorder of autophagy. Hum. Mol. Genet..

[123] Kwiatkowski T.J., Bosco D.A., Leclerc A.L., Tamrazian E., Vanderburg C.R., Russ C., Davis A., Gilchrist J., Kasarskis E.J., Munsat T. (2009). Mutations in the FUS/TLS gene on chromosome 16 cause familial amyotrophic lateral sclerosis. Science.

[124] Neumann M., Mackenzie I.R., Cairns N.J., Boyer P.J., Markesbery W.R., Smith C.D., Taylor J.P., Kretzschmar H.A., Kimonis V.E., Forman M.S. (2007). TDP-43 in the ubiquitin pathology of frontotemporal dementia with VCP gene mutations. J. Neuropathol. Exp. Neurol..

[125] Vance C., Rogelj B., Hortobagyi T., De Vos K.J., Nishimura A.L., Sreedharan J., Hu X., Smith B., Ruddy D., Wright P. (2009). Mutations in FUS, an RNA processing protein, cause familial amyotrophic lateral sclerosis type 6. Science.

[126] Neumann M., Bentmann E., Dormann D., Jawaid A., DeJesus-Hernandez M., Ansorge O., Roeber S., Kretzschmar H.A., Munoz D.G., Kusaka H. (2011). FET proteins TAF15 and EWS are selective markers that distinguish FTLD with FUS pathology from amyotrophic lateral sclerosis with FUS mutations. Brain.

[127] Neumann M., Roeber S., Kretzschmar H.A., Rademakers R., Baker M., Mackenzie I.R. (2009). Abundant FUS-immunoreactive pathology in neuronal intermediate filament inclusion disease. Acta Neuropathol..

[128] Schwartz J.C., Cech T.R., Parker R.R. (2015). Biochemical Properties and Biological Functions of FET Proteins. Annu. Rev. Biochem..

[129] Lagier-Tourenne C., Polymenidou M., Hutt K.R., Vu A.Q., Baughn M., Huelga S.C., Clutario K.M., Ling S.C., Liang T.Y., Mazur C. (2012). Divergent roles of ALS-linked proteins FUS/TLS and TDP-43 intersect in processing long pre-mRNAs. Nat. Neurosci..

[130] Dormann D., Madl T., Valori C.F., Bentmann E., Tahirovic S., Abou-Ajram C., Kremmer E., Ansorge O., Mackenzie I.R., Neumann M. (2012). Arginine methylation next to the PY-NLS modulates Transportin binding and nuclear import of FUS. EMBO J..

[131] Cairns N.J., Grossman M., Arnold S.E., Burn D.J., Jaros E., Perry R.H., Duyckaerts C., Stankoff B., Pillon B., Skullerud K. (2004). Clinical and neuropathologic variation in neuronal intermediate filament inclusion disease. Neurology.

[132] Munoz D.G., Neumann M., Kusaka H., Yokota O., Ishihara K., Terada S., Kuroda S., Mackenzie I.R. (2009). FUS pathology in basophilic inclusion body disease. Acta Neuropathol..

[133] Dormann D., Rodde R., Edbauer D., Bentmann E., Fischer I., Hruscha A., Than M.E., Mackenzie I.R., Capell A., Schmid B. (2010). ALS-associated fused in sarcoma (FUS) mutations disrupt Transportin-mediated nuclear import. EMBO J..

[134] Ticozzi N., Vance C., Leclerc A.L., Keagle P., Glass J.D., McKenna-Yasek D., Sapp P.C., Silani V., Bosco D.A., Shaw C.E. (2011). Mutational analysis reveals the FUS homolog TAF15 as a candidate gene for familial amyotrophic lateral sclerosis. Am. J. Med. Genet. B Neuropsychiatr. Genet..

[135] Urwin H., Josephs K.A., Rohrer J.D., Mackenzie I.R., Neumann M., Authier A., Seelaar H., Van Swieten J.C., Brown J.M., Johannsen P. (2010). FUS pathology defines the majority of Tau- and TDP-43-negative frontotemporal lobar degeneration. Acta Neuropathol..

[136] Ravenscroft T.A., Baker M.C., Rutherford N.J., Neumann M., Mackenzie I.R., Josephs K.A., Boeve B.F., Petersen R., Halliday G.M., Kril J. (2013). Mutations in protein N-arginine methyltransferases are not the cause of FTLD-FUS. Neurobiol. Aging.

[137] Paloneva J., Kestila M., Wu J., Salminen A., Bohling T., Ruotsalainen V., Hakola P., Bakker A.B., Phillips J.H., Pekkarinen P. (2000). Loss-of-function mutations in TYROBP (DAP12) result in a presenile dementia with bone cysts. Nat. Genet..

[138] Rademakers R., Baker M., Nicholson A.M., Rutherford N.J., Finch N., Soto-Ortolaza A., Lash J., Wider C., Wojtas A., DeJesus-Hernandez M. (2011). Mutations in the colony stimulating factor 1 receptor (CSF1R) gene cause hereditary diffuse leukoencephalopathy with spheroids. Nat. Genet..

[139] Wong T.H., Chiu W.Z., Breedveld G.J., Li K.W., Verkerk A.J., Hondius D., Hukema R.K., Seelaar H., Frick P., Severijnen L.A. (2014). PRKAR1B mutation associated with a new neurodegenerative disorder with unique pathology. Brain.

